# Postoperative feeding practices and nutritional intake in patients with head and neck cancer undergoing surgery with flap tissue transfer reconstruction: a scoping review

**DOI:** 10.1186/s12885-026-15973-9

**Published:** 2026-04-16

**Authors:** Florence Cook, Ashwa Saeed, Roganie Govender, Cecilia Vindrola-Padros, S Ramani Moonesinghe

**Affiliations:** 1 University College London, London, UK; 2 University College London Hospitals NHS Foundation Trust, London, UK

**Keywords:** Postoperative, Nutrition, Oral feeding, Head and neck cancer, Enhanced recovery after surgery, Reconstructive, Flap

## Abstract

**Introduction:**

Surgery with flap reconstruction is commonly utilised in the treatment of head and neck cancer (HNC). Following surgery, patients are made nil-by-mouth (NBM) and receive enteral tube feeding until they can recommence oral intake. Recent reviews have focussed on whether ‘early oral feeding’ (EOF) as part of enhanced recovery after surgery is beneficial. Limited literature has focussed on how patients are transitioned from tube to oral intake and nutritional implications. This review aims to map the evidence on postoperative feeding practices and nutritional intake of patients with HNC undergoing flap surgery.

**Methods:**

Titles, abstracts and subsequent full-text articles were screened from six databases: Embase, Medline, Scopus, Cochrane, CINAHL, Web of Science and grey literature by two independent researchers who both extracted data. A third reviewer was consulted when required. Inclusion criteria: all articles reporting on postoperative feeding practices in HNC flap surgery (RCTs, observational studies, case reports/series, guidelines). Findings were synthesised and presented using a narrative description approach.

**Results:**

After searching 5093 citations and 405 full-texts, 36 articles were included. Enteral feeding tubes were either intraoperative nasogastric tube or gastrostomy pre-, intra- or postoperatively with feeds ideally commencing within 24 h of surgery. Gastrostomy placement was informed by disease, treatment and socio-demographic factors including age, tumour stage/site, prior/adjuvant radiotherapy and surgery extent. The timing/type to oral feeding was mainly surgeon-led ± speech and language therapy (SLT) and varied from postoperative day 1 (sterile water ± fluids ± smooth puree ± solid/semi-solid diet) to postoperative day 20 (fluids progressing to soft diet). EOF was defined as ≤5days and delayed/traditional feeding as >5days or after a 6–12 day nil-by-mouth period. Nutritional adequacy was mainly assessed by dietitians or nurses and thresholds varied between 60 and 100% of estimated nutritional requirements.

**Conclusion:**

Postoperative feeding practices in HNC flap surgery vary and are associated with unit culture and clinician preference. Further prospective studies are required to identify factors associated with optimal timing and adequacy of oral feeding and exploration of clinician perceptions/practices.

**Supplementary Information:**

The online version contains supplementary material available at 10.1186/s12885-026-15973-9.

## Background

Surgery is the mainstay of treatment for head and neck cancers (HNC) of the oral cavity subsite and often involves resection of critical structures for speech and swallowing. For larger tumours, flap tissue transfer with pedicle or free flap is the preferred method of reconstruction due to superior functional outcomes [1].

Following surgery, patients are placed ‘nil by mouth’ (NBM) and receive enteral tube feeding until they can safely recommence oral intake. Postoperative feeding practices vary and include the timing and type of enteral feeding tubes placed, and how patients are transitioned off tube feeding and back onto oral intake. Enteral feeding tubes typically used are naso-gastric tubes or gastrostomies. Gastrostomies are utilised when enteral feeding is required for a prolonged period. Some centres will place these prophylactically depending on factors such as preoperative malnutrition and/or dysphagia and the anticipated impact of surgery and adjuvant treatment on swallowing function) [2]. The timing and type of oral feeding introduced after surgery also varies. Many centres adopt a traditional approach where a 6–12 day NBM period is employed, as it is theorised that introducing oral feeding earlier can increase the risk of complications such as development of an orocutaneous fistula (OCF) [3, 4]. Some centres have started to assess if oral feeding can be introduced earlier, known as ‘early oral feeding’ [5–7] owing to benefits including accelerated recovery and reduced length of hospital stay (LOS), without increased complications. International ERAS (Enhanced Recovery After Surgery) HNC consensus guidelines recommended that decision making for introduction of oral feeding should be led by individualised needs, acknowledging that variation exists in functional deficit following surgery for HNC [8].

Preoperative malnutrition and postoperative underfeeding are associated with poorer surgical outcomes. This includes reduced wound healing, increased development of complications, longer hospital stays, poorer quality of life and increased morbidity and mortality [9–12]. Optimising nutrition is therefore paramount. It has been reported that nutritional deficits are common in patients undergoing flap surgery for HNC affecting 40% of patients in the acute postoperative period [11]. Limited literature exists that focusses on how patients are transitioned off enteral tube feeding and onto oral intake and the impact on postoperative nutritional adequacy. Previous literature indicates that early oral feeding and individualised nutritional intervention may help ameliorate these effects, under the guidance of a multidisciplinary team including dietetic expertise [11, 13]. Preliminary feedback from our patient and public involvement group indicated that patients often felt rushed when transitioning from tube to oral intake, and those who received naso-gastric tube feeding reported tubes were removed prematurely, and at a time when they lacked confidence with eating/drinking after surgery.

### Study rationale

Two recent systematic reviews have explored the impact of early oral feeding following free-flap reconstructive surgery for HNC on postoperative outcomes including development of orocutaneous fistula and LOS [14, 15]. A scoping review is currently registered which aims to define the term ‘early oral feeding’ in HNC [16]. No reviews have been registered or published that focus on postoperative feeding management and nutritional intake/adequacy including how patients are transitioned from tube to oral feeding, the types of enteral feeding tubes placed and the timing and type of diet texture commenced.

### Aims and objectives

This review aimed to map what the postoperative feeding practices and nutritional intake are for patients with HNC undergoing surgery with flap reconstruction. The specific aims were to identify the evidence on the following concepts and clarify these concepts:

1. How patients are transitioned from tube to oral feeding following surgery with flap-tissue reconstruction for head and neck cancer with consideration to:

- The timing and type of dietary textures patients are transitioned onto.
- The type of enteral feeding tubes placed and duration of enteral feeding.

2. Adequacy of nutritional intake in patients when transitioning from tube to oral feeding following surgery with flap reconstruction in the acute postoperative phase.
3. Areas requiring further enquiry.

## Methods

Protocol and registration: The Joanna Briggs Institute (JBI) guidelines for scoping reviews [17] was followed: (1) Define the review question (2) Determine the inclusion and exclusion criteria (3) Search strategy (4) Evidence screening and selection (5) Data extraction (6) Data analysis (7) Presentation of the results. An *a priori* protocol was developed and the review was registered with Open Science Framework (2/9/24) (doi:10.17605/OSF.IO/2E38C) [18]. Ethical approval and informed consent was not required due to the nature of scoping review methodology. Results have been reported in accordance with the scoping review extension of the Preferred Reporting Items for Systematic Reviews and Meta-Analyses (PRISMA-ScR) checklist.

### Define the research question

The Population, Concept, Context (PCC) framework has been utilised to develop the research question: *What are the postoperative feeding practices and nutritional intake of patients with head and neck cancer transitioning from tube to oral feeding after undergoing reconstructive flap surgery?*

#### Population

Adults aged ≥ 18 years old with head and neck cancer (HNC) undergoing surgery with flap tissue transfer reconstruction.

#### Concept

Postoperative feeding practices and nutritional intake including type of enteral feeding tube and how/when patients are transitioned off tube feeding and onto oral intake with consideration to impact on nutritional intake/adequacy.

#### Context

Postoperative settings that cover the acute postoperative period (30days) including hospitals, outpatient clinics in the acute/community setting.

### Determine the inclusion criteria

Inclusion and exclusion criteria are detailed in Table 1.

**Table 1:** Inclusion and exclusion criteria

Study characteristics	Inclusion criteria	Exclusion criteria
Evidence sources	-Randomised controlled trials -Observational studies (including case reports/series, case-control, cross-sectional, audits, service evaluations and surveys) and qualitative studies -Reviews -Grey literature e.g. conference abstracts, guidelines and blogs	-Case reports/series with < 10 patients (and/or < 10 oral cavity/oropharyngeal subsite)*
Population	-Studies on adult humans ( ≥18 years old) with HNC (oral cavity and oropharyngeal subsite) -Postoperative patients who have undergone surgery with flap tissue transfer reconstruction (free, pedicle and local flaps) -Primary cancer surgery or surgery for recurrent disease post radiotherapy and/or chemoradiotherapy with curative intent -Where mixed population groups occurred, and not possible to separate; if the majority (≥ 75%) of the population met the inclusion criteria, these should be included*	-Animal studies -Children/adolescents (< 18 years old) -Non oral cavity or oropharyngeal subsite (e.g. larynx, nasopharynx, hypopharynx) - Laryngectomies/ laryngopharyngectomies -Non cancer cases (e.g. benign, osteoradionecrosis (ORN) or trauma) -Patients who underwent primary closure/no flap -Secondary reconstruction -Palliative surgery / non-curative intent
Concept	-Postoperative feeding practices and nutritional intake in patients -This includes studies focussing on type/timing of oral intake commencement, type/timing of enteral feeding tube placement, duration of enteral feeding and practices for removal, nutritional intake in the acute postoperative period with focus on transition from tube to oral feeding and adequacy of nutritional intake, patient and healthcare professionals’ experiences or involvement in/of postoperative feeding practices -Studies reporting feeding/nutrition within the acute postoperative phase (30 days)	-Patients were not made NBM after surgery for any duration and required postoperative enteral tube feeding -Non-HNC contraindication to commencing oral intake e.g. gastric comorbidities -For studies focussed on nutritional intake – nutritional intake cannot be interpreted i.e. from graphs or has not been reported in context of individualised estimated nutritional requirements -For studies focussed on type and timing of oral intake - type/timing not reported and/or cannot be interpreted (i.e. from graphs) or insufficient detail about feeding -For empirical studies - where postoperative feeding/nutrition was not a main focus of the article* -Studies focussed outside the acute postoperative phase (e.g. preoperative/prehabilitation or beyond 30 days postop/long-term feeding outcomes) or unclear postoperative period / unclear whether measures within the 30 days acute postoperative period
Context	-Postoperative setting e.g. hospitals/outpatient postoperative clinics (single or multicentre) -Any country	-
Language	-Abstracts and full texts in English language	-Full text not available in English language
Year	-No limit applied	-

*Deviation from original protocol during full text review

### Search strategy

The search strategy was developed in conjunction with a librarian. Six electronic databases were searched: Medline (Ovid interface, 1946–2024) Embase Classic (Ovid interface, 1947–2024), Scopus (1970–2024), Cochrane (1998–2024), Web of Science (Core collection,1900–2024) and CINAHL: Cumulative Index of Allied Health Literature (EBSCOhost, 1937–2024). Grey literature was searched through google scholar, Open Access Theses and Dissertations, charities, NHS and relevant society websites and abstract books using PRISMA guidance [19]. We searched the following clinical trial registries: *ClinicalTrials.gov*, ISRCTN Current Controlled Trials (www.controlled-trials.com), Australian New Zealand Clinical Trials Registry (www.actr.org.au).

The search strategy comprised four concepts that were derived from the research question, these included 1. “head and neck cancer”; 2. “surgical flaps’; 3. nutrition, comprising, enteral, feeding, swallowing and dietary intake, and 4. postoperative period. For each concept, we compiled an extensive list of synonyms and related terms which were linked together with the operator OR. The AND operator was used to link the four different concepts. Where appropriate, subject headings terms were also used. The search strategy for Medline (Ovid interface) is available in Supplementary File 1. The final search (across all databases) was carried out on 18th October 2024. Backward citation of reference lists of included articles and forward citation using Scopus and google scholar was conducted (December 2024).

We imposed no date restrictions on any of the searches. All languages were included in the search result; non-English full texts and animal studies were excluded during the screening process.

### Evidence screening and selection

The results of the search were exported to data management software *Covidence* (Veritas Health Innovation, Melbourne, Australia). Duplicates were removed through Covidence duplicate identification strategy and manually where applicable. Data screening and extraction of titles, abstracts and subsequent full articles were screened by two independent researchers for inclusion (FC and AS). To ensure reliability between reviewers, eligibility criteria was discussed and piloted prior to screening titles/abstracts and full-texts. A threshold of ≥ 75% agreement was set during the piloting and reviewers met frequently to discuss any discrepancies and subsequent modifications to the eligibility criteria with the team. Inter-rater reliability was assessed using Cohen’s kappa statistic; title/abstracts: 85% agreement κ_*c*_ *=* 0.53 (moderate agreement) full-texts: 99% agreement κ_*c*_ *=* 0.94 (near-perfect agreement). A third reviewer (RG) was consulted when a lack of consensus occurred.

Following screening of titles and abstracts, deviations from the a priori protocol were agreed amongst the research team:

- Case reports and articles with *n* < 10 (or < 10 of the population of interest) would be excluded when resolving conflicts at the title/abstract stage and full-text review due to the high volume of these evidence sources retrieved.
- In empirical studies with mixed population groups (e.g. cancer with benign/ORN and/or oral cavity with laryngeal subsites), if it was not possible to separate the groups, papers were only included if the majority (threshold set at ≥ 75%) of the sample studied met the population inclusion criteria (i.e. ≥ 75% were cancerous and/or oral cavity/oropharyngeal subsite). For guidelines and reviews under the umbrella of head and neck reconstructive flap surgery, if findings were applicable to population of interest (e.g. cancer and oral cavity/oropharyngeal subsite), these were included.
- For empirical studies, where postoperative feeding/nutrition were not a main focus of the paper (e.g. studies reporting on generalised surgical outcomes and included limited detail on feeding outcomes), it was agreed that these would be excluded due to volume retrieved, as they provided little evidence beyond the papers that focussed on this concept predominantly, and the concepts were also covered within the guidelines and reviews included.

### Data extraction and analysis

Results are reported in accordance with the Preferred Reporting Items for Systematic Reviews and Meta-Analyses for scoping reviews (PRISMA-Scr) [20].

A data collection tool was developed and piloted, customised from the JBI template to extract relevant data from included studies [21]. Key information collected included: country and context, publication year, design, methods, aim/objectives, study findings and outcomes. A narrative description approach was used to synthesise and present the findings. Quality appraisal or risk of bias was not undertaken, which is consistent with the principles and guidance of scoping review conduct [21].

## Results

**Fig. 1:**
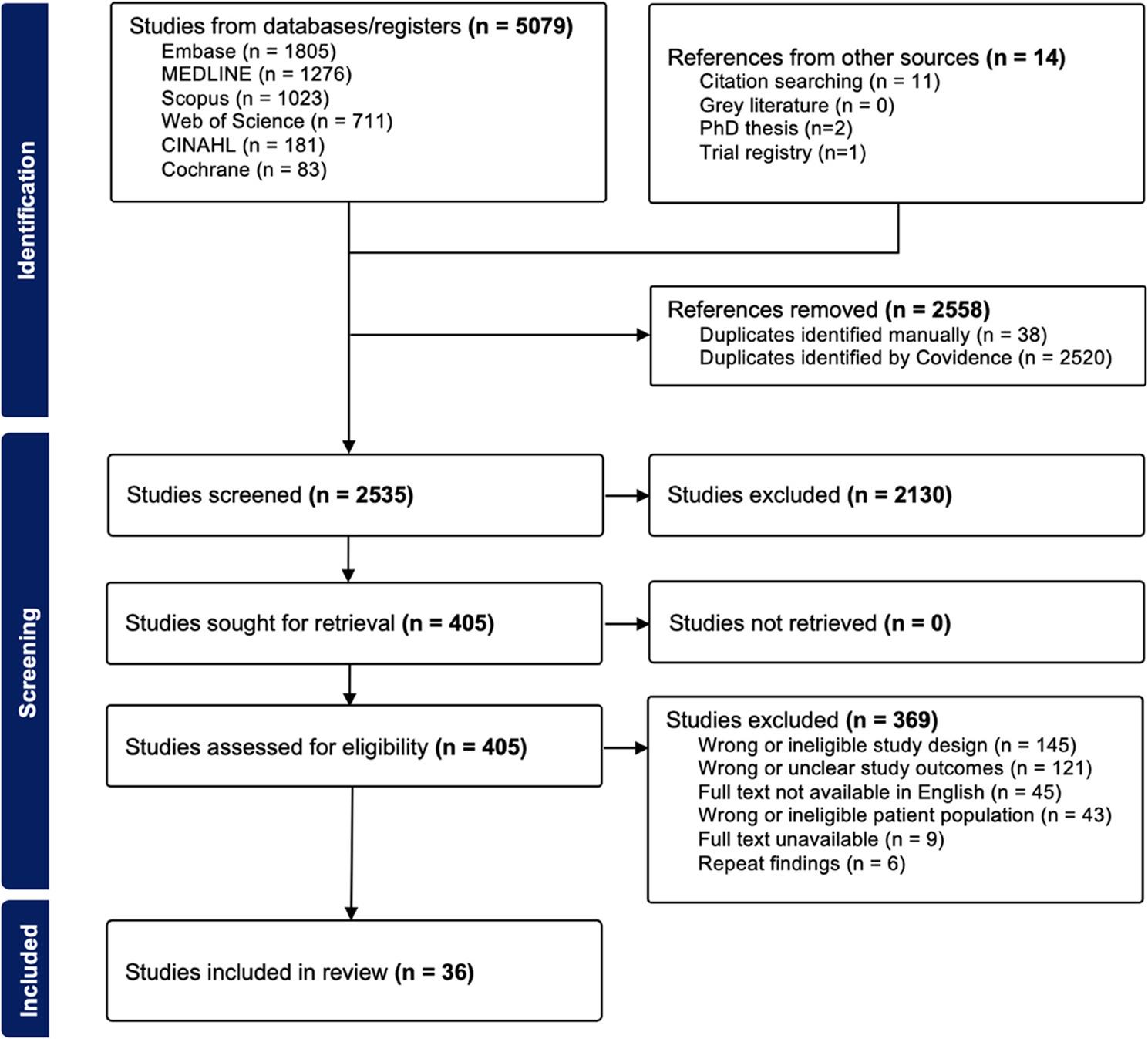
PRISMA flow diagram of the scoping review process

A total of 5093 citations were retrieved and imported into *Covidence*. Following deduplication, 2535 titles/abstracts and 405 full-texts were screened, and 36 included, delineated in Fig. 1 (PRISMA flow diagram). One paper fell slightly short of the ≥ 75% population threshold (with 69% cancer) inclusion criteria [22]. Given the relevance of this paper to the research question, and inclusion within two recently published systematic reviews with aligned concepts, it was agreed by the research team that a deviation from the protocol would be made to include this reference. Two papers and one conference abstract reported mixed HNC subsites [5, 23, 24]. The lead authors were contacted via e-mail and followed up on three further occasions to retrieve details of the disease subsites to assess if they reached the ≥ 75% threshold. A response was received for one of the articles confirming the majority of subsites were oral cavity/oropharyngeal, but no further details were provided on the breakdown [24]. No response was retrieved for the two other studies. A pragmatic decision was made to include these studies, due to expertise in the research team about structure of UK HNC services: both were conducted in oral and maxillofacial cancer units in the UK, and it was anticipated that the majority would likely meet the population criteria, as these services predominantly treat oral cavity/oropharyngeal cancer cases. Two PhD theses [25, 26] were retrieved, but as the published records associated with these were identified [11, 27] as full-text journal publications both theses were therefore excluded.

Overview of the studies: *What are the postoperative feeding practices and nutritional intake of patients with HNC transitioning from tube to oral feeding after undergoing flap-surgery?*

The 36 studies included in this review are summarised in Table 2 in narrative form of the key findings of studies and suggested areas for further inquiry [2, 4–8, 11, 14, 15, 22–24, 28–51]. Three of the studies had 2 records and both records were used for data extractions: Kerawala et al. [5], had a letter to the editor [52]. For Zhang et al., two published articles were retrieved [48, 53] and for Dean et al., two published articles were retrieved [14, 54]. 

**Table 2:** Overview of the studies and key findings related to overall research question: What are the postoperative feeding practices and nutritional intake of patients with HNC transitioning from tube to oral feeding after undergoing flap-surgery?

Study reference	Country and context	Aim and objective(s)	Study design/ type of evidence and date of data collection	Population, diagnosis and treatment (including prior surgery and/or chemo/radiotherapy)	Key findings and conclusions related to review question and objective 3) suggested areas for further inquiry
Barlow et al. (2024), Early feeding after free flap reconstruction of the oral cavity: A systematic review and meta-analysis [15].	USA (authors); Review findings: New Zealand, USA, UK, China	‘To evaluate the effect of timing of oral intake on rates of fistula formation, free flap failure and hospital length of stay in patient following free flap reconstruction of the oral cavity’.	Systematic review (November 2022)	*n* = 1085 from 5 studies; mean ages 48.3–62.9 years; males and females (% unspecified) Diagnosis: Oral cavity defects (majority squamous cell carcinomas, cancers and/or sarcomas, benign, ORN, trauma, and ‘others’ unspecified) Treatment: Free flap tissue reconstruction Prior treatment: not studied	In correctly selected patients, early oral feeding (≤ POD5) following oral cavity free-flap reconstruction is significantly associated with reduced LOS without increasing rate of OCF or flap failure. Limited number of studies with some possessing high-risk of bias. Further prospective trials are required to increase confidence in the non-inferiority of early feeding in this group and include functional outcomes to assess its benefits.
Dort et al. (2017), Optimal perioperative care in major head and neck cancer surgery with free flap reconstruction. A consensus review and recommendations from the Enhanced Recovery After Surgery Society [8].	International panel of experts representing Canada (lead author), Australia, Sweden, Switzerland and USA	‘To provide a consensus-based protocol for optimal perioperative care of patients undergoing head and neck cancer surgery with free flap reconstruction’.	Consensus guideline (March 2015)	Guideline applicable to mixed HNC surgery with free-flap tissue reconstruction (including/applicable to population subsites of interest) Prior treatment: not studied	Variation in perioperative care and evidence base in HNC free flap population. Clinical evaluation of the recommendations and further research is required in this population. Limited studies have explored timing of oral feeding or enteral nutrition support after surgery in this patient group.
Nurkkala et al. (2021). Causes of nutrition deficit during the immediate postoperative period after free flap surgery for cancer of the head and neck [11].	Finland (University Hospital)	‘To evaluate the adequacy of nutrition after free flap surgery for HNC during the immediate in-hospital recovery and to discover factors associated with inadequate nutrition delivery’.	Retrospective longitudinal single centre cohort study (2008–2018)	*n* = 218 (age/sex unspecified) Diagnosis: HNC: Oral cavity/tongue (42.6%). Maxilla (10.6%). Mandible (16.5%). Larynx (10.1%). Skin melanoma (8.3%). Buccal mucosa (9.2%). Parotid gland (2.3%). Lymphoma (0.5%). Treatment: Free-flap tissue reconstruction: RFFF (35%). ALT (31.7%). LD (1.8%). Scapula (4.3%). Fibula (15.1%). Lateral arm (6.0%). Other (5.5%). -Tracheostomy (78%). -Neck dissection: Bilateral (15.1%). Unilateral (59.2%). Nil (25.7%). Prior treatment: not mentioned	Underfeeding is common during the immediate postoperative period in HNC flap patients. Surgical complications are associated with inadequate nutrition delivery. Earlier oral food intake was associated with increased nutrition delivery. Nutrition support often failed to provide adequate nutrition to meet energy demand. Oral food intake is the most effective way to commence nutrition postoperatively in patients with undergoing free flap. Further prospective research, should explain the possible relationship between these proposed means and adequate nutritional delivery among patients with HNC undergoing free-flap.
Coyle et al. (2016). Enhanced recovery after surgery (ERAS) for head and neck oncology patients [28].	United Kingdom (University Hospital)	‘To describe the development of an ERAS protocol for people undergoing surgery for head and neck cancer’.	Service improvement (2014–2015) including prospective cohort study of adherence to ERAS protocol developed	*n* = 31 (age/sex unspecified) (adherence to protocol sub-study) Diagnosis: ERAS protocol for mixed HNC including population of interest Treatment: Surgery with free flap reconstruction ± tracheostomy and neck dissection. Prior treatment: not mentioned	ERAS for HNC surgery involves an MDT approach, facilitates greater patient involvement and improved outcomes, including reduced LOS.
Stewart et al. (2023). Predictors of gastrostomy tube placement in patients with head and neck cancer undergoing resection and flap-based reconstruction: systematic review and meta-analysis [2].	USA (authors); Review findings: Canada, USA, Japan, Germany, The Netherlands, France)	‘To establish predictors of gastrostomy tube placement among patients with head and neck cancer undergoing surgical resection with immediate pedicled or free flap-based reconstruction’.	Systematic review and meta-analysis (Data collection inception- April 2020)	*n* = 1112 (age 54.14 ± 3.13 years, 77% male across eleven studies. Diagnosis: Mixed HNC excluding temporomandibular joint, lip, buccal, skin, dura/skull base, nasopharynx, hypopharynx, nasopharyngeal tumour locations. Treatment: Surgery with free or pedicled flap reconstruction excluding total glossectomy, laryngectomy, jejunal and ALT reconstruction flap subtypes due to only single studies with these specific categories. Prior treatment: prior radiotherapy considered	Preoperative identification of those who will require a gastrostomy tube that have HNC undergoing flap reconstruction is a clinical challenge. Risk factors for gastrostomy include advanced tumour stages and prior radiation therapy. More RCTs are required to confirm these findings and develop a decision-making algorithm to guide an MDT approach to caring for these patients.
Guidera et al. (2013). Early oral intake after reconstruction with free flap for cancer of the oral cavity [6].	New Zealand (General Hospital)	‘To investigate the impact of early oral intake on postoperative outcome of patients having resection’.	Retrospective cohort study/consecutive case series? (dates not mentioned but paper accepted 2012).	*n* = 54 (*n* = 29 early group, age 60 ± 15.4years, *n* = 25 late group age 62 ± 26.5 years). Diagnosis: Oral cavity cancer T1/2 (39%) T3/4 (61%). Mandible (13%). Maxilla (6%). Buccal mucosa (6%) FOM (24%). Hard palate (9%) Retromolar trigone (9%). Tongue (anterior 2/3) (33%). Treatment: Resection and reconstruction with free flap: Ulna forearm (61%). RFFF (4%). DCIA (19%). Other (17%). No neck dissection (15%) Ipsilateral (65%) Bilateral (20%) Tracheostomy (86% of the early and 92% late group; duration early group 6(2) days and late group 8.3(6). Prior treatment: Prior RT or surgery were excluded	EOF does not result in increased postoperative complications (including OCF) but is associated with significantly shorter LOS. Prospective larger randomised study is required to verify findings and to further assess potential benefits of EOF, including consideration to extent of resection.
Navarro Vila et al. (1989). Enteral nutrition in patients with tumours of the head and neck [29].	Spain (OMFS surgical service in general hospital).	Aim not specified but inferred to evaluate the use of alternative nutrition support, such as enteral nutrition in patients with head and neck tumours undergoing surgery.	Retrospective cohort study/case series (1985–1986).	*n* = 30 (aged 53.4(38–78) years 67% males). Diagnosis: HNC in undernourished patients Treatment: Reconstructive surgery for oral cavity or oropharynx defects. Tongue (27%). Lower Lip (7%). FOM (17%) Maxilla (13%). Retromolar trigone (10%). Cheek (7%) Tonsil (10%). Soft palate (10%). 100% T3 or T4 tumours (T3N0M0 to T4N2bM0). Treatment: Myocutaneous flaps, Osteomyocutaneous flaps and/or free anastomosed myofascial temporal flap. Prior treatment: not mentioned	Administration of EN in patients undergoing HNC surgery avoids postoperative malnutrition and infection (by food remaining in the oral cavity).
Dawson et al. (2017). Factors affecting orocutaneous fistula formation following head and neck reconstructive surgery [30].	United Kingdom (University Hospital)	‘To identify complications with a particular focus on the formation of fistulaes following head and neck reconstructive surgery’.	Retrospective single centre cohort study (May 2013-Oct 2014).	*n* = 102 (age/sex not specified) Diagnosis: HN defects; Cancer (86.3%) ORN (7.8%) Ameloblastoma (3.9%) Trauma (2%). T0-2 (47%) T3/4 (48%) NA (5%). Treatment: Surgery with a free or pedicled flap. Laryngectomy, pharyngectomy or reconstruction of the pharynx alone excluded 98% oral cavity/oropharyngeal: mandibulectomy/rim resection/mandibulotomy (48%). maxillectomy (20%), total/partial glossectomy (21%), total parotidectomy (1%) FOM (3%) rhinectomy (1%), soft palate and oropharynx (1%). Full thickness cheek resection (1%). Other (5%). Fibula flap (38%). ALT (15%). RFFF (33%). Scapula (10%). Pec major (4%). Nasolabial flap (1%). Prior treatment: 21% had previous surgery with adjuvant chemoradiotherapy. 5% had previous chemoradiotherapy and presented with new primary tumours or ORN.	The day oral intake started, and formation of fistula was not statistically significant, but 8/11 of patients who developed a fistula started oral intake before day 8. In this unit, based on the findings of this study, patients who have previously had CRT do not commence oral intake until the 8th postoperative day. More prospective findings needed to explore this concept. Whilst a RCT is gold standard for assessing a relationship between complications and day oral intake is initiated, confounding variables such as dysphagia and tracheostomy weaning warrant consideration. A cohort study may be more pragmatic and provide an appropriate level of evidence. Limited literature exists on oral diet initiation outside of primary surgery without previous CRT or composite resections.
Stramiello et al. (2021). Timing of postoperative oral feeding after head and neck mucosal free flap reconstruction [31].	USA (University Hospital and Medical Centre)	‘To better understand the impact of early feeding (EF) on swallowing function and fistula rates in patients with free flap reconstruction of upper aerodigestive tract defects’.	Retrospective cohort study in 2 centres (March 2016-April 2019)	*n* = 104 (*n* = 24 early and *n* = 80 late feeding groups). 65.4% male. Age 62.81 ± 14.18 and 62.14 ± 13.59 years early and late groups respectively. Diagnosis: HN defects: Cancer (82.7%) (*n* = 21 EF and *n* = 65 LF groups). Those presenting with a fistula requiring a free flap for fistula were excluded. Oral cavity (75%; 23 early, 55 late groups). Oropharynx/hypopharynx (17.3%, 0 early and 18 late groups). Mixed aerodigestive (4.8%, 0 early and 5 late group). Maxilla (2.9%, 1 early and 2 late groups). Treatment: Resection with free flap reconstruction (types not specified but paper indicates this included fibula, ALT, osteo and fasciocutaneous RFFF and Rectus). Prior treatment: Preop chemo (25%, 3 early and 23 late groups) Preop radiotherapy (27%, 1 early and 27 late groups)	Early oral feeding was not associated with adverse outcomes and/or increased fistula formation in properly selected patients and was associated with improved functional swallowing outcomes earlier. Timing of oral feeding depended on institution, surgeons’ experiences and confidence, tissue quality, prior radiation (an important consideration but did not preclude early feeding) and/or other treatments, comorbidities, baseline swallow function, and reconstructive characteristics. Future, multi-institutional prospective larger studies are needed, combining patient, provider and system factors that interplay with surgical recovery to understand optimal timing of postoperative oral feeding. RCTs of postoperative feeding pathways would be challenging to design and carry out, as not all patients may be suited to aggressive postoperative feeding pathways. Studies should identify patients suitable for early oral feeding and if they have better swallow function outcomes.
Imai et al. (2024). Enhanced recovery pathways for head and neck surgery with free tissue transfer reconstruction [32].	Japan (Head and Neck Cancer surgery centres)	‘To describe the recommended components of the ERAS pathways in HNC surgery, especially in free flap tissue reconstruction and outline the outcome obtained by the ERAS pathways based on results of systematic reviews and meta-analysis and investigations within institutions’.	Literature review (date of search not specified)	Review findings applied to HNC surgery including those undergoing free flap reconstruction/ population of interest. Prior treatment: no mentioned	ERAS pathways are gaining traction in head and neck surgery and are associated with reduced LOS, but efficacy remain uncertain. Future research should focus on improving the quality of postoperative patient recovery and satisfaction using patient reported outcomes, to determine whether ERAS is beneficial. Use of perioperative immunonutrition requires further examination as there is no definitive evidence in head and neck surgery for its usefulness.
Brady et al. (2021). Early postoperative feeding: an investigation of early functional outcomes for oral cancer patients treated with surgical resection and free flap reconstruction [33].	UK (Head and Neck Unit, Cancer Hospital)	‘To evaluate the early functional swallowing outcomes and complication rates using a detailed and replicable early feeding protocol for patients undergoing free tissue transfer reconstructive surgery for oral cavity cancer in the primary setting’.	Service evaluation (consecutive series Jan 2018-Dec 2019)	*n* = 29 (age 59.5 (24–88) years, 58.6% male) Diagnosis: Oral cancer Tongue (34%). Maxilla (21%). Mandible (7%). FOM (17%). Buccal mucosa (7%). Stage IV (68%) Treatment: Free flap surgery: ALT (38%), Fibula (24%), RFFF (21%), MSAP (17%). Prior treatment: not mentioned	Early oral feeding protocols have potential for shorter LOS and earlier swallowing rehabilitation. Ongoing variation in the UK and elsewhere, thus further prospective data are required to define and evaluate an evidence-based protocol for early feeding in the postoperative setting following resection of oral cavity with free flap reconstruction, including use of patient reported outcome measures.
Cook et al. (2022). Oral feeding commencement following free or pedicled flap reconstruction for oral cancer: Experience from a tertiary referral centre [4].	UK (Head and Neck Unit, tertiary centre, University Hospital)	‘To review current practice for initiating oral diet postoperatively, examine the time to postoperative oral diet initiation post flap reconstruction and determine if there is a relationship between number of days after surgery oral diet was commenced and LOS’.	Retrospective service evaluation / consecutive case series (Dec 2018 to Nov 2020	*n* = 34 (age 60.8 ± 12 years; 52.9% male). Diagnosis: Oral cancer. Disease stage 1/2 (35%) 3/4 (65%). Buccal mucosa (5.9%), Mandible (32.4%), Tongue (44.1%). Maxillary sinus/palate (5.9%), Sublingual gland (2.9%). FOM (5.9%), Maxilla (2.9%). Treatment: Free or pedicled flap surgery. Maxillectomy (12%), Mandibulectomy/rim (35%), Partial/hemi glossectomy (44%), FOM (6%), Buccal (3%). Flaps: RFFF (62%). Fibula (8%). ALT (25%). Scapula (4%). Pec major (29%), FAMM (20%). Nasolabial (10%). Submental (30%). Tracheostomy (62%) days 9(3–48). Neck dissection (88.2%, *n* = 30 lateral, *n* = 4 bilateral). Excluded laryngectomies and primary closure Prior treatment: Excluded patients who had previous radiotherapy.	Traditional approaches for postoperative oral feeding practices existed in the study unit. Weak relationship observed between earlier commencement of oral feeding and shorter LOS. Further research should identify factors predictive of safe commencement of oral feeding in HNC post flap surgery, to identify the most appropriate candidates for early oral feeding. Qualitative research is needed, to explore attitudes and practices amongst the MDT (including HNC surgeons) alongside patient experiences.
Denholm et al. (2022). Factors determining postoperative length of stay and time to resumption of feeding following free flap reconstruction for oral cancer [34].	UK (Acute general hospital)	‘To benchmark the time to resumption of oral feeding following free flap reconstruction for oral cancer and identify factors associated with time to resumption of oral feeding’.	Retrospective review (March 2015 to October 2020)	*n* = 100 (aged 62 (35–82) years, 70% male). Diagnosis: Oral cancer Stage 2 (54%), Stage 3/4 (46%) Mandibular mucosa (24%) tongue (23%) FOM (19%), oropharynx (11%), maxilla (8%) other (15%). Excluded non-cancer Treatment: Free flaps 64% composite and 36% soft tissue. Radial, fibula, MSAP, ‘other’ – unspecified used. 50% bilateral neck dissection, 48% unilateral and 2% no neck dissection. 16% had tracheostomy at time of surgery Prior treatment: not mentioned	Tracheostomy, female gender and increasing age were strongly associated with delays in resuming some types of oral feeding. ICCU stays of two or more days was associated with a longer time to resuming free fluids. This information can help target the use of enhanced recovery programmes and cost analyses.
Eley et al. (2012). A review of postoperative feeding in patients undergoing resection and reconstruction for oral malignancy and presentation of a pre-operative scoring system [35].	UK (University Hospital)	‘To review the benefits and complications of PEG and nasogastric (NG) feeding in patients and develop a scoring system to aid selection of the most appropriate tube’.	Retrospective review (Jan 2006 to 2010) as an audit of clinical practice	*n* = 144 (age 62(29–88) years, 61% male). Diagnosis: Oral cancer Mandible (29%). Maxilla (2%) FOM/ventral tongue (13%). Lateral/posterior tongue (40%). Buccal mucosa/cheek/flap (13%). Tonsillar fossa, pharynx, soft palate (3%). Treatment: Flap reconstruction ALT (10%) RFFF (67%), Fibula (8%), LD (5%), Scapular (1%), ALT + fibula (3%), Radial + fibula (7%). Prior treatment: states treatment for recurrent tumour in *n* = 9.	The tool developed in this study can be used to select correct tubes in 92% of patients. Key factors that result in prolonged nutrition support include site and size of tumour, method of reconstruction and extent of planned radiotherapy postoperatively. Further prospective review and validation of the tool is required but can be used to assist clinicians with difficult decisions.
Bater et al. 2017. Enhanced recovery in patient having free tissue transfer for head and neck cancer: does it make a difference? [36].	UK (OMFS unit, District General Hospital)	‘To design an ERAS programme to reduce the duration of stay in hospital for patients who have free tissue transfer for head and neck cancers’.	Prospective cohort study (Jan 2013 for 3 years prospective cohort compared to a historical cohort treated in 2012 who didn’t have a formal recovery programme)	*n* = 140 (100 ERAS, 40 historical TRAS (traditional recovery) patients), matched patients (*n* = 72). Age 67(59–78) TRAS and 59(49–67) ERAS groups. Sex not specified. Diagnosis: head and neck defects FOM/tongue (42%), mandible (34%), maxillectomy (16%), lip/cheek (4%), soft palate (5%). T1/2 (55%). T3/4 (31%). Benign/NA (14%). Treatment: Free flaps. Soft tissue *n* = 71, bone *n* = 69. RFFF (69%), fibular (16), ALT (12%), Lat dorsi (2%), DCIA (1%), RFFF + bone (1%). Tracheostomy (58%). Prior treatment: not mentioned	ERAS programme is safe and effective, potentially reducing LOS in HNC free flap reconstructive surgery and cost savings of £400/patient/day = £1600/patient. No significant differences were found for complications or readmissions. More extensive data and a large multicentre trial to is required to establish best practice for use of ERAS programmes and evidence reductions in LOS.
Højvig et al. (2022). Enhanced recovery after microvascular reconstruction in head and neck cancer - A prospective study [37].	Denmark (University Hospital, tertiary healthcare facility)	‘To present results after the implementation of the enhanced recovery protocol for microvascular reconstruction after head and neck cancer and discuss options for further development and progress in this challenging patient population’.	Prospective cohort study (June 2019 and Dec 2020) of ERAS group compared with a historical cohort of TRAS patients (2014–2016)	*n* = 88 (*n* = 30 ERAS and *n* = 58 TRAS group). Age 64.5(43–85) and 62.3(31–84) years, 65% male. Diagnosis: mixed HNC, ORN excluded. 91% cancer. Subsites: oral cavity (92%), sinus and nasal cavity (8%). Treatment: Resection + free flap Fibula (41%), Lat dorsi (25%), ALT (20%), RFFF (8%), Fibula + LD/ALT (6%). Patients undergoing laryngectomy excluded. Tracheostomy - ERAS (10%) and TRAS (90%), time to ambulation reduced from 7.9 to 4.4 days. Prior treatment: *n* = 36 (prior surgery *n* = 8, RT *n* = 10, both *n* = 18).	Introduction of ERAS in HNC with microvascular reconstruction is safe, improves recovery and reduced LOS without increasing the risk of infection, surgical site complications, flap survival or readmission. Further improvements in ERAS programmes include prehabilitation programmes and early identification of patients at risk of nasogastric tube dependence to prevent prolonged hospitalisation facilitating discharge with a NGT or PEG tube with an outpatient regime for restarting oral intake. A multicenter study is required to validate the protocol including demographic differences in this heterogenous population.
Hwang et al. (2023). Intraoperative enteral nutrition feeding in free-flap healing after reconstruction surgery for head and neck cancers [38].	Taiwan (University Hospital, single tertiary care centre)	‘To investigate the beneficial outcomes of intraoperative enteral feeding in free flap regeneration after extended head and neck cancer resection’.	Pilot RCT double-blind placebo-controlled trial (April 2020 to April 2022)	*n* = 56 (*n* = 28 in fasting and *n* = 28 in feeding groups); Age 52.9 ± 9.3 and 56.2 ± 7.9 years fasting and feeding groups. 98% male. Diagnosis: Oral HNC. Buccal (52%), Tongue (20%), Gingival (21%), Pharyngeal (5%) Lip (2%). Treatment: Free flap reconstruction: ALT (77%), Fibula (4%), RFFF (20%). Tracheostomy (58.9%, *n* = 19 fasting and *n* = 14 feeding groups). Prior treatment: Preop radiotherapy *n* = 2/3.6% (1 in each group).	Enteral feeding during HNC reconstructive surgery significantly reduced the occurrence of wound dehiscence, edge necrosis and LOS without increasing pulmonary aspiration. Perioperative enteral feeding is a safe and effective approach to improve perioperative protein catabolism and proinflammatory reactions which enhances early flap recovery and wound healing. Future research should assess if intraoperative enteral feeding benefits are volume dependent.
1 reference with 2 reports: (1) Dean et al. (2024). Early oral feeding and its impact on postoperative outcomes in head and neck cancer surgery: a meta-analysis [14] (2) Dean et al. (2024). Does the timing of oral feeding affect the fistulisation risk among head and neck cancer patients undergoing free flap reconstruction? [54]	Egypt (lead author), Saudi Arabia, Peru, USA, India, Lebanon (co-authors); Review findings: New Zealand, USA, UK, China.	‘To compile the evidence on the association between early initiation of oral feeding and postoperative complications (e.g. fistula formation, seroma development, and flap failure) and LOS following HNC reconstructive free flap surgeries’.	Systematic review and meta-analysis (Inception- August 2023)	*n* = 1097 (384 early and 713 late feeding) across 5 studies. Aged 51.5 ± 14.22 to 62.9 ± 13.66 years. Sex not reported in all studies but in 4 ranged: 57% to 65.3% male. Population: Mixed HNC. Sites included mandible, maxilla, buccal mucosa, FOM, hard palate, retromolar trigone, tongue. 1 study included pharynx and larynx albeit majority oral cavity (Stramiello et al.) [31]. Treatment: Free flap (ulna forearm, RFFF, DCIA, fibula, scapular, ALT, osseocutaneous RFFF, iliac crest). No differences in tracheostomy rates in early versus late feeding. Prior treatment: no differences in prior chemo/radiotherapy in early versus late feeding groups.	Early oral feeding is significantly associated with shorter LOS and doesn’t increase complications in patients with HNC undergoing free flap surgery. Surgeons should consider implementing early oral feeding protocols in this patient group without worrying about the risk of fistula, even amongst those who have had preoperative chemoradiotherapy. Future research should encompass various age groups (as majority of patients were > 60years, reflecting the HNC population), histopathology, cancer site locations, free-flap types and postoperative complications to investigate the optimal timing for postoperative oral feeding more comprehensively.
Kinzinger & Bewley (2017). Perioperative care of head and neck free flap patients [39].	Authors (USA, university hospital).	‘To identify and discuss the most recent literature on the optimal perioperative care of head and neck free flap patients’.	Literature review. Date of literature review searches not reported	Diagnosis: Review findings applied broadly to head and neck surgical patients undergoing free flap reconstruction including cancer. Prior treatment: Not mentioned	Research into postoperative care suggests nutrition is a factor that can improve wound outcomes, decrease LOS and costs. There is a low level of evidence supporting individual ERAS recommendations in head and neck perioperative care. Further research is required to define and implement optimal comprehensive care regimens for this complex population. This includes describing the most effective means of perioperative nutritional intervention.
Vincent et al. (2019). Perioperative care of free flap patients [40].	Authors (USA, head and neck surgical units).	‘To review current literature surrounding care of head and neck free flaps patients to provide a guide to surgeons’.	Literature review. Date of literature review searches not reported	Diagnosis: Review findings applied broadly to head and neck surgical patients undergoing free flap reconstruction including cancer. Review findings also given guidance on care of tracheostomy Prior treatment: Not mentioned	Head and neck free flap care is multifaceted and optimally managed using an individualised approach with support from the multidisciplinary team. Evidence to support immunonutrition varies with mixed results.
List et al. (2023). Enhanced recovery after surgery, current, and future considerations in head and neck cancer [41].	Authors (USA, head and neck surgical unit/cancer centre)	‘To provide a comprehensive summary of the current and evolving areas of multidisciplinary optimisation at all phases of care for the HNC patient, from diagnosis to discharge and to develop a list of evidence-based recommendations that can be implemented in head and neck surgical practices’.	Literature review. GRADE system used to assign strength of evidence. Date of literature search: inception to September 2022	Diagnosis: Review findings applied broadly to head and neck cancer surgery with free flap reconstruction. Search included HNC, head and neck surgery, oral cavity cancer, total laryngectomy and variations of these terms. If literature in HNC was lacking, high-level evidence of non-head and neck surgery was included if deemed applicable across fields. Treatment: search included head and neck surgery, enhanced recovery after surgery, total laryngectomy, free-flap reconstruction and variations of these terms. 18 aspects of perioperative care were included. Prior treatment: not mentioned	ERAS protocols for patients undergoing head and neck surgeries requires thorough collaborations from the multidisciplinary teams and is still a developing process but reduces complications, shortens LOS and reduces costs whilst providing superior perioperative care in HNC surgery. While there is increasing research in HNC, many recommendations are generalised from other surgical fields. Future research will strengthen and add new principles to a comprehensive HNC ERAS protocol.
McAuley et al. (2015). Early feeding after free flap reconstruction for oral cancer [7].	United Kingdom (OMFS surgery unit, teaching hospital, Northern Ireland)	‘To evaluate postoperative outcomes of early oral feeding in 10 consecutive patients who had free tissue transfer after resection of oral cancer. Hypothesised that early feeding prevents the muscles from forgetting the habit of swallowing and has a positive and beneficial effect on rehabilitation after operations of this nature’.	Retrospective review (convenience sample). Data of data collection not specified	*n* = 10; (aged 68(53–79) years. Diagnosis: oral cancer T1/2 (80%), T3/4 (20%). Sites: alveolus/mandible (30%), buccal mucosa (20%), FOM (20%), Tongue (30%). Treatment: Free flap: RFFF (70%), Fibula (30%). 100% neck dissection- ipsilateral (70%) and bilateral (30%) 100% had a tracheostomy (duration 1.5 ± 0.53 days) Prior treatment: not mentioned	Early oral feeding does not adversely affect outcome and/or rehabilitation in patients with oral cancer undergoing flap surgery and favours adoption in postoperative recovery programmes due to shorter hospital stay. A larger prospective study is required to confirm and evaluate these findings.
Niziol et al. (2024). A rapid recovery protocol for head and neck oncology patients undergoing resection, free flap reconstruction and tracheostomy: a feasibility study [24].	United Kingdom (University Hospital)	‘To investigate the feasibility of a rapid recovery protocol (RRP) to further enhance perioperative care in conjunction with the ERAS protocol. This included describing a patient selection methods and potential cost savings’.	Prospective cohort study with comparison to matched controls (July 2019 to March 2021; halted Feb 2020 to July 2020 due to covid-19) RRP cohort compared to standard postoperative matched controls.	*n* = 91 (*n* = 26 RRP group, aged 56(27–71), 70% male with *n* = 10 not completing the RRP pathway and *n* = 65 standard postoperative care group (2019) of which *n* = 9 were matched controls. Diagnosis: HNC; subsite not specified, contacted lead author who advised 100% were oral cavity cases, ASA grade 1–3. Treatment: Free flap and tracheostomy (RRP group - Fibula (46%), RFFF (35%), ALT (15%), scapula (4%). Prior treatment: not mentioned	RRP group were decannulated 5 days earlier and discharged 7 days earlier compared matched controls, which amounted to cost savings of £9955/patient, £159,280 total. A multidisciplinary approach to patient selection for RRP can reduce the need for intensive care unit admission in patients with HNC undergoing free flap and decrease LOS and tracheostomy use. Further larger studies are required recruiting into this RRP.
Chandu et al. (2003). Percutaneous endoscopic gastrostomy (PEG) in patients undergoing resection for oral tumours: A retrospective review of complications and outcomes [42].	Australia (teaching hospital)	‘The aims of this study were to review the complication rates in a consecutive series of patients undergoing resection and reconstruction for oral tumours and to assess and compare their weight and body mass index as indicators of nutritional status preoperatively and postoperatively’.	Retrospective review (1992–2001)	*n* = 49 (aged 6(3–86) years, 57% male) Diagnosis: Oral cancer, 90% oral cavity (Stage 1/2 (19%), Stage 3/4 (81%), 6% mandibular ameloblastoma, 2% ORN. Treatment: Reconstructive surgery; type of flap not specified. 59% adjuvant radiotherapy Prior treatment: 1 patient initially underwent radiotherapy as the primary treatment modality but required surgery later.	Due to benefits of PEG placement in patients with oral cancer, unit policy is that those undergoing major oral tumour resection have a PEG placed, preferably at the time of resection and by an experienced endoscopist to support nutrition in the postoperative and radiotherapy phases to maximise convalescence and recovery. Findings limited by retrospective study design, and lack of control group.
Müller-Richter et al. (2017). Nutrition management for head and neck cancer patients improves clinical outcome and survival [43].	Germany (authors, OMFS unit, University hospital)	‘To review the literature and give an overview of stress metabolism, nutrition, and strategies for perioperative nutrition management so that the reader can conduct evidence-based nutrition management in HNC patients’.	Literature review. Date of literature review searches not reported.	Diagnosis: Review applies to head and neck cancer reconstructive surgery/ tumours of the upper aerodigestive tract including reconstruction with flap tissue/population of interest. Prior treatment: not mentioned	Existing data support nutrition management for patients with head and neck cancer for improved treatment course and survival.
Yamaguchi et al. (2024). Effect of oral intake initiation-establishment interval on hospital stay after oral cancer surgery [44].	Japan (University hospitals − 1 for surgery and 1 for rehabilitation)	‘To clarify the effect of the period between early initiation of oral intake to established oral intake on LOS in patients receiving gradual dysphagia rehabilitation’.	Retrospective study (2015–2021). 2 groups based on duration between early initiation and establishment of oral intake: shorter period group < 2 days, long-period group > 3 days	*n* = 106 (*n* = 50 short, *n* = 56 long period group). Aged 62.9 ± 15.6 and 68.5 ± 11.5 years. 51% male. Diagnosis: Oral cancer T1/2 (28%) T3/4 (72%). Tumour subsite: tongue (46%), oral floor (7%), buccal mucosa (7%), gingiva (39%), hard palate (1%). Treatment: Resection, neck dissection and free flap in 84% of patients (*n* = 41 early and *n* = 48 late groups). Type of flap unspecified. Prior treatment: Perioperative chemo/radiotherapy in 36.8% (*n* = 15 early and *n* = 24 late group). Doesn’t detail proportion pre-op.	Reducing LOS requires both earlier initiation and early established oral intake. The period between initiating oral intake and established oral intake is associated with LOS and swallowing function at discharge in patients with oral cancer after surgery. Effective nutritional management and dysphagia rehabilitation is necessary during hospitalisation and after discharge. Multicentre larger prospective studies are required to clarify the effects of dysphagia rehabilitation on in-hospital outcomes.
Wu et al. (2022). Timing of oral feeding in patients who have undergone free flap reconstruction for oral cancer [45].	China (University hospital)	‘To design a nasogastric removal plan for patients with or without tracheostomy according to swallowing function and oral intake and perform a parallel randomised clinical trial to determine the safety of the methods and their effectiveness in facilitating early oral feeding’.	Prospective randomised controlled trial (May 2021 to December 2021) Patients assigned into one of four groups, each *n* = 32: a non-tracheostomy control and intervention group, a tracheostomy control and intervention group.	*n* = 128 (aged 49.2 ± 12.8, 47.3 ± 17.9, 53 ± 12.4, 56.5 ± 11.8, 59% male) Diagnosis: Oral cancer Surgical sites: Buccal (13%), Maxilla (15%), Mandible (31%), Tongue (13%), Mouth floor (9%), root of tongue (7.0%), Palate (10%), lip (1%). Treatment: Resection + free flap. RFFF (20%), Fibula (31%), ALT (25%), iliac crest (23%). Prior treatment: Patients with prior radiotherapy were excluded and those with high tension at the suture of the intraoral wound (determined by the surgeon) or dysphagia (due to other causes such as stroke).	Early oral intake after surgery does not increase wound complications and pneumonia or adversely affect oral intake of patients. It can help minimise pharyngeal pain and reduce LOS in patients with a tracheostomy. The limiting aspect of oral intake after surgery is not wound healing, but the safety risk caused by aspiration, and risk of the insufficient food intake due to oedema and wound pain. Ensuring safety and effectiveness can facilitate early oral feeding in this patient group.
1 reference, 2 records (1) Kerawala et al. (2021). The impact of early oral feeding following head and neck free flap reconstruction on complications and length of stay [5] (2) Singh et al. (2021). An urgent need for early oral feeding following head and neck reconstruction with oral defects [52].	United Kingdom (Head and Neck Unit, tertiary referral centre, Cancer Hospital)	‘To evaluate the outcomes of early feeding on a cohort of patients undergoing free flap reconstruction of oral defects with particular emphasis on post-operative complications and compare them with a cohort of our patients managed with the more traditional approach of delayed feeding’.	Prospective cohort study (dates of data collection not provided)	*n* = 400 (*n* = 200 early and *n* = 200 late feeding groups). Age: early feeding 60.2(18–89) years and late feeding 65.6(18–85). Male to female ratio was 2.1:1 and 2.6:1 early and late feeding groups. Diagnosis: Oral defects; Early feeding group: Cancer (66–70%) ORN (23–25%). Missing information for 5–11%. Assuming at least 70/95 and 66/89 were cancer in late and early feeding groups respectively, this equates to 74% overall cancer. Subsites not specified - authors emailed, no response. Likely mainly oral cavity/oropharynx as oral cancer title. Letter to editor also requested subsite data. Treatment: Free tissue transfer. Composite flap in 37.5% late and 36.5% early feeding. RFFF in 33% late and 25% early feeding groups, ALT 15% late and 32% early feeding groups. A small unspecified number underwent double flap. Tracheostomy in 6% late and 20% early groups. Bilateral neck dissection in 6% late and 10% early groups. Prior treatment: Nearly half (in both groups) underwent prior surgery or chemo/radiotherapy.	Early oral feeding after reconstruction with free tissue transfer does not lead to increase in local complications and reduces LOS. Findings challenge the ‘arbitrary’ NBM period in traditional practices, even for high-risk patients such as smokers, those with previous RT, where some authors recommended remaining NBM for 8 days. Cause and effect are not yet established between early oral intake and early discharge, but delaying oral intake may contribute to the stress on both patients and health resources. Letter to editor indicates that early oral feeding should also be considered in other forms of reconstruction such as pedicled and local flaps and from their experience, early sterile water in pedicled flaps help mucosal wound edges to be clean and promotes faster healing, maintains oral hygiene and sensation of well-being for the patient. Urge for RCTs for concept of early oral feeding to confirm these findings.
Rana et al. (2023). Resumption of oral feeding following reconstruction of oral cavity defects using the submental artery perforator flap (SAIPF) [46].	USA (University)	‘To demonstrate that the SAIPF for oral cavity reconstruction is associated with acceptable functional outcome and identify factors associated with delayed oral feeding postoperatively’.	Retrospective study (2014–2023); conference abstract - poster	*n* = 54 (aged 74(52–98)years, 59% male. Diagnosis: Oral cavity defects- Cancer (93%) Benign (6%) ORN (2%). Tongue (41%), mandibular gingiva (39%), FOM (9%) buccal mucosa (7%), lip/commissure (4%). pT1/2 (78%) pT3/4 (16%), N0 (76%), N1 (16%), N2/3 (8%). Treatment: Surgery with submental artery perforator flap (SAIPF). Neck dissection unilateral (87%) bilateral (13%). Tracheostomy (29.6%). Prior treatment: RT (5.6%).	In older patients with oral cavity defects following surgery, with SAIPF some may need tube feeding at the time of discharge. This is often temporary, and most return to an oral diet following dysphagia rehabilitation. Increasing tumour stage, FOM and tongue sites are strong predictors for increased time to oral feeding following SAIPF of the oral cavity. No standard algorithm for oral feeding commencement exists but some centres do this within 5 days.
McAfee et al. (2019). Enhancing the recovery of head and neck oncology patients through early feeding [23].	United Kingdom (OMFS surgery unit, teaching hospital Northern Ireland)	‘The aim is that 100% of patients to be NBM for 2 days or less by June 2018’.	Prospective audit; conference abstract BAOMS (dates not given but indicates the target for change was June 2018)	*n* = 30, no demographic details. Diagnosis: HNC - subsites not clear, likely majority oral cavity/oropharynx as BAOMS conference. Emailed lead author, no response. Treatment: RFFF or fibula flap (other reconstructions excluded). Prior treatment: not mentioned	Reducing the NBM period has no detrimental effect on flap reconstruction success and improves patient rehabilitation and utilisation of hospital resources.
Main et al. (2017). Early oral intake and length of stay following free flap reconstruction of the oral cavity [47].	United Kingdom (HNC surgical unit, District General Hospital)	‘To assess the impact of an early oral intake policy on length of stay following reconstruction for oral cavity cancer’.	Not stated but appears to be a retrospective audit (Jan 2013 to Dec 2016). Conference abstract BAOMS.	*n* = 41 (aged 54.8 years, 59% male). Diagnosis: States tumour stage/site was recorded in methods, inferring all were cancer, oral cavity subsite Treatment: Methods infers all had a free flap, results states RFFF (76%). Tracheostomy (5%)	Early oral intake is safe and reduces LOS without additional complications or morbidity.
Le et al. (2022). Does early oral intake after microvascular free flap reconstruction of the oral cavity lead to increased postoperative complications? [22]	USA (OMFS unit, University Hospital)	‘To identify associations between timing of oral intake and study variables, identify associations between postoperative complications and study variables, identify whether timing of feeding is associated with postoperative complications and LOS and identify variables that influence postoperative complications’.	Retrospective cohort study (Jan 2014 to Dec 2019)	*n* = 415 (aged 58(14–88) years, 61% male) Diagnosis: Oral cavity mucosal defect. Cancer (69%) Benign (16%) ORN/osteonecrosis (11%), Trauma (5%). Sites: buccal mucosa (6%), FOM (6%), mandible (52%), maxilla (19%) and tongue (17%). Treatment: Resection + free flap. External facial defects with minimal oromandibular involvement and isolated hypopharyngeal defects following total laryngectomy were excluded. Prior treatment: 16% prior chemo/radiotherapy.	Authors state that findings verify early oral feeding (< 5 days) is safe and associated with decreased LOS. High risk variables included defect location, extent of neck dissection and presence of a tracheostomy or gastrostomy tube which should be considered prior to initiating oral intake. Further studies should incorporate patient reported QOL measures and clarify what types of patients would benefit from early oral feeding within an ERAS protocol versus traditional pathways
1 reference; 2 records: Zhang et al. (2022 and 2024). Effects of personalised swallowing rehabilitation in patients with oral cancer after free flap transplantation: A cluster randomised controlled trial. [48, 53]	China (University hospital)	‘To evaluate the effects of personalised swallowing rehabilitation on oral cancer after free flap transplantation patients on swallowing function, nutritional status, and quality of life’.	Randomised controlled trial (August 2021 to January 2022)	*n* = 68 (*n* = 34 intervention and *n* = 34 control group). Age 52.66 ± 12.61 years, 66% male. Diagnosis: Oral cancer: buccal mucosa (16%), Oral floor (7%), Tongue (22%), Palate (16%), gingiva (38%). Stage 1/2 (35%) 3/4 (54%) Unclear (10%). Treatment: Resection and free flap: RFFF (21%), Fibula (32%), Iliac bone (18%), ALT (29%). Neck dissection: Unilateral (68%), Bilateral (19%), Nil (13%). Tracheostomy (43%), Prior treatment: Previous radiotherapy excluded.	Personalised swallow rehabilitation can improve swallowing function and ability, promote early NGT removal and recovery of oral feeding, improve nutritional status and QOL. Long-term effects of rehabilitation could not be evaluated as 1 month follow-up measures taken due to possible impact of adjuvant radiotherapy. Multicentre study is required to validate findings and study the effect of oral exercise training on tongue function.
Wu et al. (2023). Early swallowing training after free flap surgery in oral cancer: A randomised controlled trial [49].	China (University hospital)	‘To compare and analyse the effects of swallowing training on postoperative swallowing function, weight loss rate, QOL and postoperative complications’.	Prospective randomised controlled trial (Feb 2022 to August 2022)	*n* = 121 (*n* = 59 control and *n* = 62 intervention groups). 51.5 ± 13.8 and 49.5 ± 14.2 years. 69% male. Diagnosis: Oral cancer. Buccal (15%), FOM (5%), Tongue (23%), Maxilla (17%), Mandible (26%), Palate (15%), Stage T1/2 (50%) 3/4 (48%) Unclear (2%) Treatment: Free flap surgery Fibula (31%), RFFF (20%), ALT (32%), Iliac crest (17%). Neck dissection: unilateral (60%), Bilateral (17%), Nil (24%). Tracheostomy (42%).	Early postoperative swallowing training in patients with oral cancer free flaps improves postoperative swallow function, QOL, nutritional status, and shortens NGT indwelling time. Further studies should focus on the impact of swallowing training on the swallowing function of patients undergoing radiotherapy after surgery.
Nesemeier et al. (2017). Evidence-Based Support for Nutrition Therapy in Head and Neck Cancer [50].	USA (University Hospital)	‘To review current nutrition literature pertaining to head and neck cancer patients and to present evidence-based strategies for nutritional support specific to this population’.	Literature review (date of lit search not specified)	Review applies to generalised HNC surgery including oral cancer and flap reconstruction/population of interest. Prior treatment: not mentioned	Nutritional optimisation of patients and predicting the need for alternative enteral access postoperatively can help minimise perioperative morbidity and improve adherence to standard treatment algorithms. Immunonutrition improves perioperative outcomes. Postoperative nutrition should be guided by a registered dietitian, using patient-specific parameters to establish nutritional requirements. Future studies should identify optimal perioperative nutritional care in HNC, as majority of the existing evidence is retrospective, single-centre or from other populations.
Khan et al. (2021). Early Feeding After Free Flap Reconstruction of Oral Cavity Defects: A Single Arm Non-inferiority Trial (NCT04787939) [51]	USA (Otolaryngology department, University Hospital); 2 sites as part of the same centre.	‘To evaluate surgical outcomes in subjects who are fed in the first days after oral cavity reconstructive surgery and compare rates of orocutaneous fistula in subjects who are allowed to eat immediately after surgery to those rates published in the literature (individuals for whom oral feeding is delayed for several days after surgery). To determine whether early feeding on postoperative day 1 is non-inferior with respect to the development of orocutaneous fistula to the standard care described in the literature’.	Prospective single-arm non-inferiority trial; registry/ protocol in recruitment (March 2021-anticipated end Dec 2025)	Target sample *n* = 89 (Adults aged ≥ 18, male or female who speak English, Spanish or Mandarin and can consent). Diagnosis: oral cavity defects (conditions include HNC) Treatment: Free tissue transfer reconstruction Prior treatment: prior major oral cavity surgery or radiation excluded and those with known history of dysphagia or with current enteral feeding needs.	This study will aim to evaluate the safety of early feeding in patients with oral cavity reconstruction who are allowed to eat by mouth on postoperative day 1. Traditionally, surgeons have opted to delay the time to oral feeding because of concern that an early oral diet may stress intraoral suture lines and lead to the development of salivary leaks. Evidence from studies evaluating oral feeding timing in laryngectomy suggests no increased risk associated with early oral feeding. Due to 50–60 oral cavity reconstructive surgeries performed at the centre per year, it is not feasible to recruit two study arms for direct comparison. The study team will also report outcomes related to wound healing, LOS, duration of enteral tube feeding, swallow evaluation, and patient reported outcomes measures.

*Abbreviations*: *HNC *head and neck cancer, *RFFF *radial forearm free flap, *ALT* Anterolateral thigh flap, *LD* latissimus dorsi flap, *DCAI* deep circumflex iliac artery flap, *MSAP* medial sural artery perforator flap, *FOM* floor of mouth, *ORN* osteoradionecrosis, *RCT* randomised controlled trial, *BAOMS* British Association of Oral and Maxillofacial Surgeons, *QOL* quality of life, *ERAS* Enhanced recovery after surgery

Studies comprised four randomised controlled trials [38, 45, 48, 49], one prospective single-arm non-inferiority trial registry (ID: NCT04787939) [51], three systematic reviews [2, 14, 15], six literature reviews [32, 39–41, 43, 50], one consensus guideline [8], four prospective cohort studies [5, 24, 36, 37], eleven retrospective studies [6, 7, 11, 22, 29–31, 34, 42, 44, 46], three audits [23, 35, 47], and three service evaluation/improvement studies [4, 28, 33]. One of the retrospective studies [46] and two of the audits [23, 47] were published conference abstracts.

**Fig. 2:**
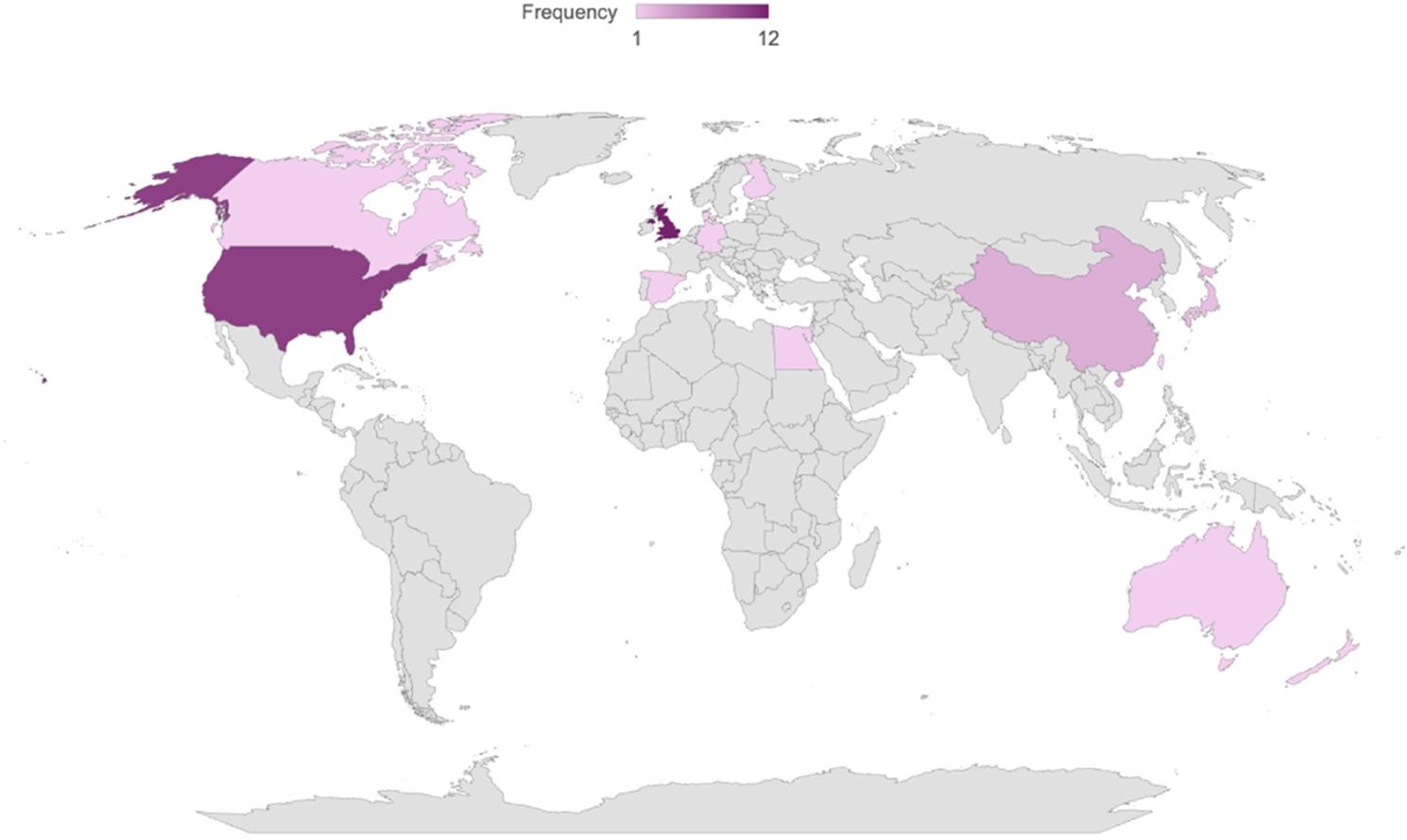
World heat map demonstrating number of studies conducted on postoperative feeding practices by country. Number of studies by country: United Kingdom (n=12), United States of America (n=10), China (n=3), Japan (n=2), Canada (n=1), Finland (n=1), New Zealand (n=1), Spain (n=1), Denmark (n=1), Taiwan (n=1), Egypt (n=1), Australia (n=1), and Germany (n=1)

Studies originated from 13 countries, as depicted in Fig. 2. The majority arose from western countries including United Kingdom (*n* = 12) and United States of America (*n* = 10) with the remaining from China (*n* = 3), Japan (*n* = 2), Canada (*n* = 1), Finland (*n* = 1), New Zealand (*n* = 1), Spain (*n* = 1), Denmark (*n* = 1), Taiwan (*n* = 1), Egypt (*n* = 1), Australia (*n* = 1), and Germany (*n* = 1). For reviews and guidelines with international authorship, the country of the lead author was recorded as the study origin.

Sample sizes ranged from *n* = 10 to *n* = 415 (single studies) and *n* = 1112 (systematic review encompassing findings of 11 studies).

The findings of the studies against the key objectives are summarised in Supplementary File/Table 3 in narrative form.

### Objective 1a: the timing and type of dietary textures patients are transitioned onto

The timing of oral feeding as defined in evidence sources or within results is summarised in Table 3.

**Table 3:** Timing and type of oral feeding commencement postoperatively

Timing of oral feeding and type of oral feeding (if specified)	Reference
POD1	[15, 41]
POD1 or 2 (sterile water)	[7]
POD1 (sips water ± smooth puree); with majority tolerating fluids	[33]
POD1 (fluids ± solid or semi-solid diet); with 30% tolerating solid/semi-solids	[5]
POD1 or 2 (clear fluids)	[23]
POD1 (liquids, not specified)	[51]
POD2 (water), POD ≥ 3 for fluids. POD3-10 ‘some oral intake’ / by discharge	[28]
POD3 (sips water)	[24]
POD3 or 4/5/7 (puree or sloppy diet)	[7]
POD3-5 (sips water ± smooth puree ± soft moist ± soft and bite sized)	[33]
POD4 (sips water ± free fluids)	[36]
POD4 (fortisips)	[24]
POD ≤ 5	[15, 22, 31, 41]
POD ≤ 5 (fluids)	[6, 54]
POD ≤ 5 (sips water)	[34]
POD5 (puree diet)	[36]
POD < 5 (ranged 1–5 days)	[46]
POD < 6	[40]
POD5 (3–7)	[11]
POD 3 (1–16) days (oral fluids)	[47]
POD ≥ 5	[22]
POD > 5	[15, 31]
POD > 5 (started on fluids and progressed to soft diet)	[14]
POD > 5 (5 days NBM followed by a few days fluids and then soft diet)	[5]
POD6 (late feeding, following a 5 day NBM period)	[6, 15]
POD ≥ 6 (half the cohort resumed oral diet on ≥ 7 days)	[46]
POD6 (earliest commenced diet)	[48]
POD6 (1–17) days (soft diet)	[47]
POD6 (5–8)	[11]
POD ≥ 8 NBM period for those with prior CRT	[30]
POD 11.9 ± 4.1 days (range 4–20)	[4]
POD 15 (± 5.1 or ± 6.2 days)	[44]
POD3-10 ‘some oral intake’ / by discharge	[28]
NBM for 10–20 days postoperatively	[31]
NBM for up to 12 days postoperatively	[33]
NBM for 6–12 days postoperatively	[4, 14, 23]
≥ 7-day NBM period postoperatively	[29]
5–7 days NBM period postoperatively	[4, 39]
NBM for 1–2 weeks postoperatively	[23]
Transition to oral diet in first 24 h ‘often impossible’ in reconstructive surgery	[43]

*Abbreviations*: *POD* postoperative day, *NBM* nil−by−mouth, *fortisips* oral nutritional supplement drink, equivalent of free fluids, *CRT* chemoradiotherapy

‘Early’ oral feeding was defined as ≤ 5 days across seventeen studies and < 6 days in one study [40]. Specifically, seven studies recommended this could start at POD1: unspecified diet [15, 41], liquids [51], fluids ± solid or semi-solid diet (with 30% tolerating solid/semi-solids on POD1 and 84% by POD3) [5], sips water ± smooth puree (as stipulated by early oral feeding protocol, with results mentioning majority managed oral fluids on POD1) [33], POD1 or 2: sterile water [7], clear fluids [23]. One study recommenced water on POD2 [28]. Five studies recommended commencement on POD ≥ 3 of: POD3: sips water [24], POD ≥ 3 of fluids [28], POD3 or 4/5/7 of puree or sloppy diet [7], POD3-5 of sips water ± smooth puree ± soft moist ± soft and bite sized diet [33], average POD3 (range of 1-16days) of oral fluids achieved [47]. Two studies reported commencement on POD4 of sips water ± free fluids [36] or ‘Fortisips’ (an oral nutritional supplement equivalent to free fluids) [24]. Ten studies reported oral feeding commencement ≤ 5 days [15, 22, 31, 41] with a range of 1–5 days [46] and 3–7 days [25] and more specifically, as fluids [6, 14], sips water [34], and puree diet [36].

Rationales for early oral feeding or early swallowing included benefits of reduced LOS [6, 7, 14, 15, 22, 23, 33, 36, 40, 47], or anticipated improved LOS [51], increased nutrition delivery and/or status [11, 48, 49], improved swallowing outcomes/rehabilitation [23, 31, 33, 41, 48, 49] or anticipated improved swallowing outcomes [51], reduced pharyngeal pain due to expedited NGT removal (45), early NGT removal [48, 49], reduced risk of infection due to promotion of salivary secretion [45], improved utilisation of hospital resources [23] and improved QOL [48, 49], or anticipated improved QOL [51]. Eleven studies, including two systematic reviews demonstrated that early oral feeding was not associated with (or reduced) complications including OCF, wound dehiscence, leak, flap failure, repeat operations and morbidity [5–7, 14, 15, 22, 31, 36, 45, 47, 49]. More specifically, of the two systematic reviews, Barlow et al., reported that early oral feeding displayed no increase in free flap failure (RD = -0.01, *p* = 0.39) or OCF (RD = -0.02, *p* = 0.06) whist LOS was significantly shorter (mean difference in days − 2.43, *p* < 0.01) [15]. Similarly, Dean et al., reported that early oral feeding was not significantly associated with postoperative fistulas (RR 0.49, *p* = 0,07), flap dehiscence (RR 0.85, *p* = 0.71), flap failure (RR 0.85, *p* = 0.67), neck haematoma/seroma (RR 0.71, *p* = 0.38) or wound infection (RR 0.45, *p* = 0.09), but was associated with a shorter LOS (MD -3.18, *p* = 0.0003) [14].

‘Delayed’, ‘traditional’ or ‘conservative’ oral feeding practices was defined > 5 days by four studies [5, 14, 15, 31], after following a 5 day NBM period [5, 14, 15] and ≥ 5 days by one study [22]. Recommended NBM period ranged from POD6-20, with a 6–12 day NBM period most frequently cited. One study indicated that the transition to oral diet in the first 24 h is often impossible in reconstructive surgery [43]. Specifically, the following studies recommended starting on POD ≥ 6: fluids for a few days and then progressing to soft diet after a 5-day NBM period [5, 14], unspecified [15, 48], average POD6 (range 1-17days) of soft diet [47] and average POD6 (range 5–8 days) [25], and POD ≥ 6 with half of the cohort resuming oral diet (unspecified) on POD ≥ 7 [46]. The following studies stipulated more stringent NBM periods; beyond 5 days. One study recommended commencement from POD ≥ 8, with a 8-day NBM period advised to avoid the risk of fistula in patients with prior CRT [30]. One study achieved oral feeding commencement an average of 11.9 days (range 4–20 days) [4], 15 days (± 5.1 and 6.2 days) [44]. NBM periods typical of traditional feeding practices were referenced as 5–7 days [4, 39], ≥ 7 days [29], 6–12 days [4, 14, 23], ≤ 12 days [33], 1–2 weeks [23] and 10-20days [31].

Rational for delayed/traditional/conservative feeding practices most commonly included concerns of developing a complication including OCF, wound dehiscence, infection and leak [29, 40], with one non-inferiority trial in recruitment measuring these outcomes [51]. These concerns were mainly theoretically derived and contributed to wariness around the potential risks of introducing oral intake amongst clinicians. However, one study reported an increased incidence of fistulas in patients with a history of chemoradiotherapy who started oral feeding before POD8 [30] or concern when “early feeding is given from the more cranial side” [32]. Furthermore, two studies reported a limited or weak relationship between timing of oral feeding with LOS (which authors report is likely due to the ability to discharge with NGTs within their units) [4, 34].

Factors supporting candidacy for early oral feeding included surgery as initial oncologic treatment (and/or no prior radiotherapy), oral cavity primary, reconstruction of the oral cavity [31], smaller primary tumours, mandible [22, 31] and buccal mucosa reconstruction, smaller skin paddle area and defect volume [22]. It is also inferred that early personalised swallowing rehabilitation results in earlier resumption to oral feeding [33, 48, 49]. Factors supporting candidacy for delayed feeding included a history of prior radiotherapy/chemoradiotherapy [30, 31]. Conversely, one study reported prior radiotherapy did not increase rates of fistulas amongst patients who received early oral feeding [5]. Other factors included poor baseline swallow, comorbidities such as hypothyroidism and diabetes (due to their association with poor wound healing) [31], tracheostomy presence/indwelling time [6, 22], increasing tumour stage, defect location (floor of mouth and tongue) [22, 46] and extent of neck dissection [22]. Age, tracheostomy and longer critical care unit stay were associated with reduced likelihood of resuming free fluids and female gender was associated with longer time to puree diet [34]. Contrary, age, defect size and proximity of a suture line to pooled saliva were not critical factors for timing to oral feeding in another study [31] or pedicled versus free flaps [4]. Several studies cited operating/attending surgeon judgement, preference, experience and confidence with attainting a watertight flap inset (to reduce the risk of fistula) and centre practices/philosophical approaches important factors that influence the timing to oral feeding [5, 7, 22, 24, 31, 32, 39, 40, 46, 51, 54]. Other healthcare professionals referenced in decision-making for timing/type of oral feeding most commonly included Speech and Language Therapy (SLT)-led assessment [5, 28, 33, 51], or SLT in consultation with the surgeon and/or dietitian [22, 24, 50, 51]. Specifically, the role of the surgeon was to assesses for risk of fistula and then following clearance of this, the SLT assesses swallowing function, and the dietitian supports with explaining the role of tube feeding and swallow/texture adjusted meals. Four studies [44, 45, 48, 49] reported the role of a swallowing specialist nurse or dentist trained in dysphagia. This was likely in place of an SLT, and these studies were all based in Japan and China. No qualitative studies were identified exploring patient or HCP experiences, perceptions or practices for timing/type of oral feeding.

**Fig. 3:**
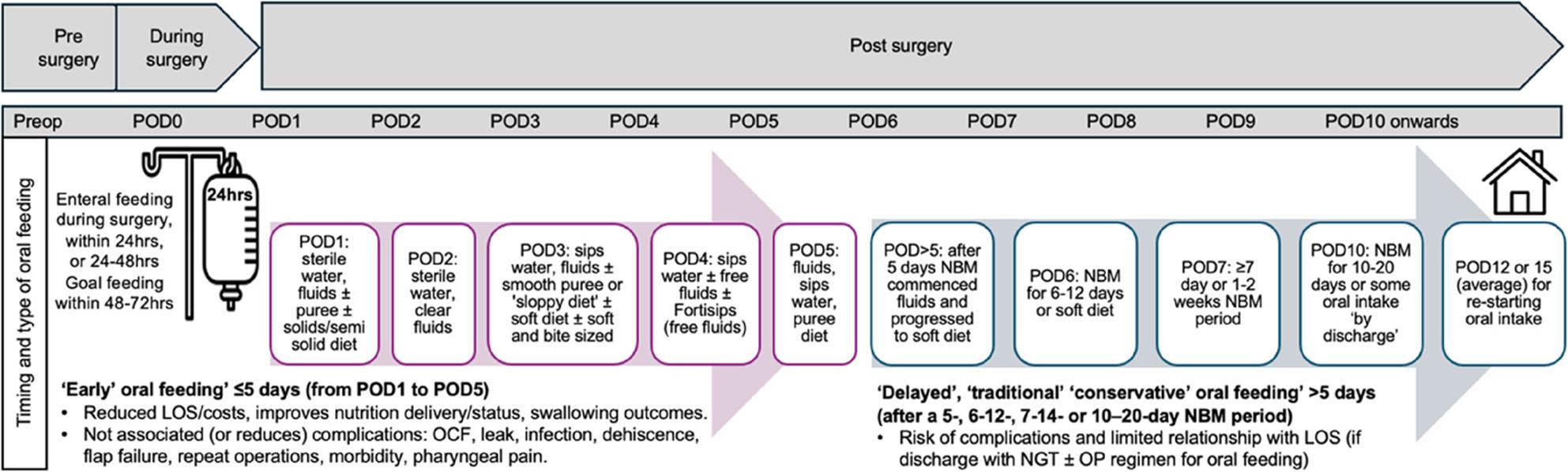
Timing/type of oral feeding commencement. Abbreviations: POD = postoperative day, OCF = orocutaneous fistula, CRT = chemoradiotherapy, LOS = Length of stay, NGT = Nasogastric tube, OP = outpatient, NBM = nil−by−mouth

These results are summarised in Fig. 3, which illustrates the timing and type of oral feeding from the evidence sources in this scoping review.

### Objective 1b: the timing/ type of enteral tube feeding and duration of enteral feeding

Route of nutrition following surgery was most commonly enteral, described as via a nasoenteric/NGT or gastrostomy tube (PEG – percutaneous endoscopic gastrostomy or RIG – radiologically inserted gastrostomy), in 15 studies [4, 6, 8, 22, 28, 30, 31, 33–37, 40, 44, 50]. Use of jejunostomy was reported in one study for a single patient, where gastrostomy was contraindicated [35]. Eight studies reported or inferred use of NGTs only [5, 7, 24, 29, 38, 45, 49, 53] and two studies reported on gastrostomy tubes only [2, 42]. In three studies, use of a feeding tube is described, but the type of tube was unspecified [11, 46, 51].

The least common route was parenteral nutrition (PN). In two guidelines, PN was only advised if there was either no enteral access or this was unsafe, or prolonged issues with feeding tolerance [8, 50]. In contrast, one review advised use of enteral nutrition ± PN [43] and two original studies cited use of IV dextrose in addition to enteral nutrition [11], or ‘fluid electrolyte replacement therapy’ for the first 48hrs followed by enteral feeds via NGT’ [29].

Timing of NGT was reported (or inferred) as intraoperative [6, 24, 35, 38, 50], by the HNC surgeon in four studies [24, 35, 38, 50]. One study specified placement by the operating HNC surgeon [38] at induction or immediately after the tracheostomy, before the operation [6], after tumour resection and before commencing free-flap [38] or at the end of the operation [35]. Two studies reported ability to discharge patients with NGT as a factor that enabled discharge and/or avoids prolonged LOS [4, 34].

Choice of tube, including decision making for gastrostomy was referred to as an MDT based approach by four studies [2, 8, 28, 33] with input from dietetics and SLT and/or based on baseline swallowing SLT assessment [22]. Placement of gastrostomy was reported in three studies as performed by gastroenterologists / endoscopists [35, 36, 50], one study as by interventional radiologists [22] and one study by the upper gastrointestinal surgeons [42].

Ten studies reported on the role of prophylactic gastrostomy [2, 4, 8, 22, 28, 33, 35, 40, 42, 50] where decisions for gastrostomy are made before surgery, at the point of diagnosis [8] or at MDT appointments [28] and are placed pre- or intraoperatively or in the immediate postoperative period. The timing of gastrostomy was reported amongst studies as an average of: 6.2 days (range 1–17) pre-op [42] 14 days (range 2–26) pre-op [35], postop day 1 or postop days 7–14 [33]. Another study reported placement by postop day 7 to enable discharge [37]. Two studies inferred a reactive approach, where gastrostomies were placed in patients who initially had NGTs, but were either unable to meet their nutritional needs orally or required prolonged nutrition support [37, 44]. Rationales for gastrostomy included: where long-term feeding (≥ 4 weeks) was anticipated due to surgery and/or adjuvant therapy e.g. radiotherapy (to the primary site and neck bilaterally) [6] and impact on worsening/causing dysphagia [4, 6, 8, 33, 35, 40], pre-op baseline swallowing function and patient preference [33] and to enable discharge or avoid delays in discharge due to post-op procedure scheduling [22, 37]. Two studies specifically studied factors associated with requiring gastrostomy in patients with HNC undergoing flap surgery [2, 35]. Risk factors for requiring a gastrostomy included advanced tumour stage (T stage 3–4) or recurrence, prior radiotherapy, age > 70 years, pre-op BMI < 18.5 kg/m^2^, American Society of Anaesthesiologists (ASA) physical status grade III-IV, alcohol consumption > 40units/week, medications > 3, intraoperative tracheostomy, total glossectomy, excision of posterior 1/3 tongue, anterior 2/3 tongue ± floor of mouth, mandibulectomy, tonsilla fossa/pharyngeal/soft palate, oropharyngeal versus segmental mandible tumour resection, and > 50% tongue resection compared to floor of mouth, ≥ 2 simultaneous flaps, fibula/anterolateral thigh/latissimus dorsi flap (with radial forearm flap associated with lower need for gastrostomy compared to rectus abdominis and latissimus dorsi and no difference found between pedicled and free flaps), adjuvant radiotherapy of more than one field.

The timing of enteral feeding was recommended and/or reported as ≤ 24 h in ten studies [8, 28, 32, 36, 39–43, 50], with one study citing a goal of 12–24 h [28] and two recommending within 24 h or at latest, 48 h for nutritionally high-risk patients [32, 50]. One study reported an advance to goal feeding within 48–72 h if tolerated, otherwise prolonged over 5–7 days [50]. One trial commenced EN via NGT intraoperatively, at a rate of 10-20 ml/hr depending on residual gastric content [38]. Another study reported use of fluids electrolyte replacement therapy for the first 48 h, after which enteral feeding via NGT could commence [29].

Noteworthy, this study was from 1989, and therefore would not reflect the updated evidence advocating early enteral feeding (≤ 24 h). The type of feeds utilised were standard polymeric [8, 38, 40] or immunonutrition [8, 32, 40, 43, 50]. Only one study advocated use of immunonutrition, citing benefits including positive effects on wound healing, infection, fistula, complications, LOS and costs [43] with remaining studies all reporting insufficient, varied, equivocate or lack of definitive evidence of benefits.

**Fig. 4:**
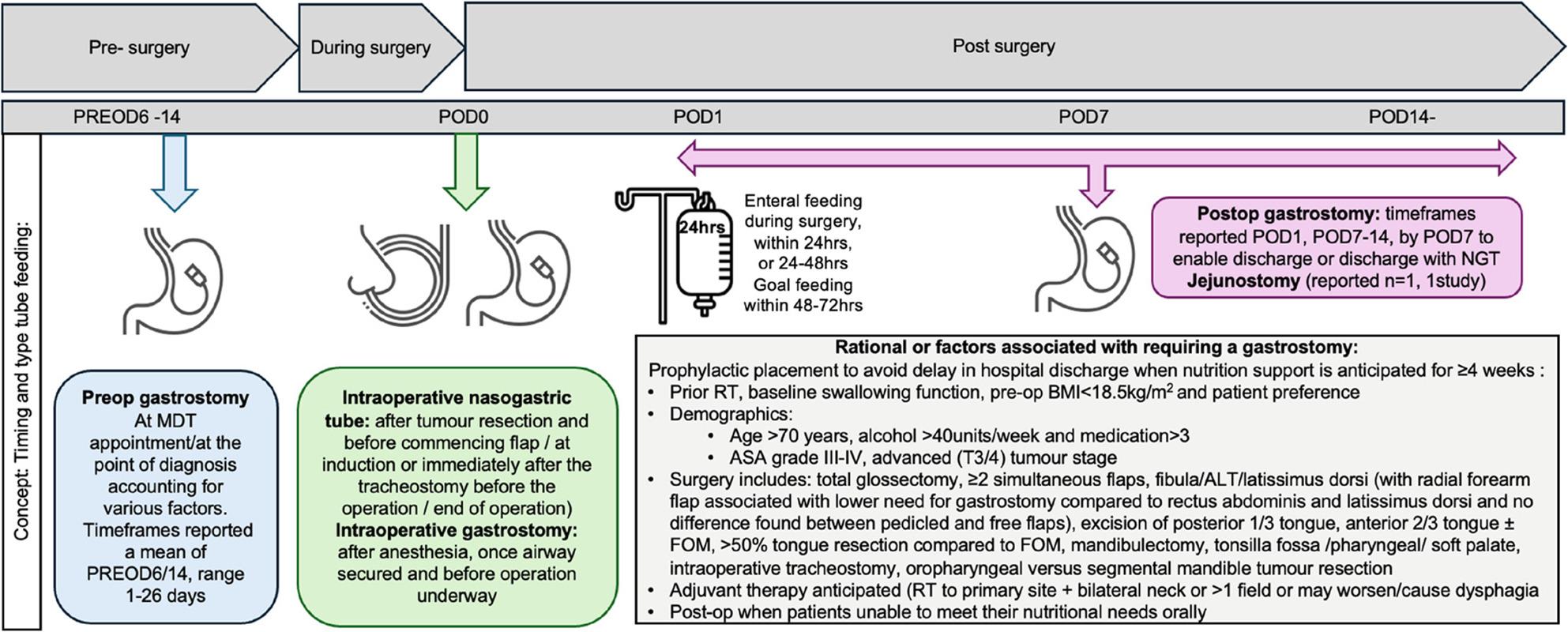
Timing/type of tube feeding. Abbreviations: PREOD = preoperative day; POD=postoperative day, OCF=orocutaneous fistula, CRT = chemoradiotherapy, LOS = Length of stay, NGT = Nasogastric tube, OP = outpatient, NBM = nil-by-mouth, ALT = anterolateral thigh flap, FOM = floor of mouth

These results are summarised in Fig. 4, which illustrates the timing and type of tube feeding from the evidence sources in this scoping review.

### Objective 2: adequacy of nutritional intake in patients when transitioning from tube to oral feeding following surgery with flap reconstruction in the acute postoperative phase

One study stipulated an aim of discharge within its ERAS protocol to be “adequate nutrition intake” [28]. Another study reported enteral feeding could be withdrawn once patients were able to maintain adequate nutrition on oral feeding alone [31]. Five studies reported on thresholds used to enable discontinuing tube feeding or removal of feeding tubes. This included 60% [11] or 75% [24] of estimated individual nutritional requirements or 100% estimated individual nutritional requirements [37, 44] or population estimates of nutritional requirements [45].

The following details and assumptions were provided to support the thresholds. In Wu et al. [45], population estimates assumed that most patients with oral cancer in China are 50-70years old and these individuals have an average weight of 59.2–70.6 kg (taken from a ‘National Physical Fitness Monitoring Bulletin’, 2014). Nutrition support guidelines for cancer in China recommend 25-30 kcal/kg/day and 1–2 g/kg/day protein which were used to determine nutritional requirements, and equated to 1480-1765 kcal and 59.2–70.6 g protein/day. In the study, intake of a 2000 ml liquid diet containing 1700 kcal and 70 g/day was assumed nutritionally adequate. NGT removal time ranged from 5 ± 2.3 days (non-tracheostomy intervention group) to 16.2 ± 13 days (tracheostomy control group), and authors report NGTs were removed as soon as daily food intake was considered adequate (equivalent to > 2000 ml), which theoretically meant a minimum 1-day period between commencing oral feeding and removing the tube. In Nurkkala et al. [11], nutritional requirements were estimated individually using ideal body weight. Energy requirements were calculated at 30 kcal/kg/day, derived from European Society for Clinical Nutrition and Metabolism (ESPEN) guidelines for surgical patients and national UK guidelines for HNC patients (with calculated average energy demand 1784 and 2060 kcal/day for the adequate and inadequate groups respectively). The rationale for 60% threshold was taken from American critical care guidelines and studies that have used this threshold in intensive care settings to depict the difference between malnutrition and satisfactory nutrition. In Niziol et al. [24], no information as provided on how nutritional requirements were calculated or the 75% of recommended calories adequacy threshold. A ≥ 75% threshold has been used in studies in colorectal cancer [55], where this has been shown to be sufficient for weight maintenance in inpatients [56]. Furthermore, ESPEN guidelines for surgery [57] and cancer [58] advise use of medical nutrition (enteral or parenteral) if patients are unable to eat adequately, thresholds set at < 50% nutritional requirements for > 1 week or 50–75% of requirements for > 2 weeks. In Yamaguchi et al., criteria for NGT removal was when 100% of estimated nutritional requirements (using the Harris-Benedict 1918 formula) was achieved orally [44].

Three studies did not specify an adequacy threshold but recommended that sufficient postoperative supplementation was aligned with an energy intake of 30-35 kcal/kg/body [43] to 40-45 kcal/kg/day [50] for nutritional repletion after surgery weight/day and a protein intake of 1.2–2 g/kg body weight/day [43], 1.2–1.5 g/kg/day or 2 g/kg/day (range 1.5–2.5 g/kg/day), due to the risk of sarcopenia in this group [50] and to promote would healing. These values included referenced ESPEN guidelines for cancer and surgery and PN and ASPEN (American Society for Parenteral and Enteral Nutrition) guidelines for critically ill patients and the 40-45 kcal/kg originated from a non-HNC specific reference. Another study recommended an average energy intake of 2090 ± 270 kcal/day using the Harris-Benedict formula with 1.3 physical activity factor and a 1.4 stress factor [29] or with a 1.5 factor for activity and stress (which equated to 30 kcal/kg/day adjusted body weight) [50].

The following studies reported on the time between commencing oral feeding to withdrawing EN. It was reported or inferred (from timing to oral feeding and duration of tube feeding) that NGTs were removed as early as the day of, or one day after oral feeding started, or pre discharge [4, 7, 28, 33, 37, 48] amongst the following studies: withdrawing EN varied from POD7 (1–9) / POD6 (3–15) with commencement of oral feeding from POD1 and protocol stipulating NGT removal from POD3-5 [33], to POD4 following commencement of puree diet on POD3/4 [7], to POD12.9 ± 5.3 following oral feeding commencement from 11.9 ± 4.1 days [4], POD13.3 (1–30) and 22.7 (1-126) in ERAS and traditional recovery (TRAS) groups respectively [37] and POD8.37 (7–10) (intervention group) and POD 17.76 (10–22) (control group), inferred oral feeding commencement POD6 [48].

The period between commencing oral feeding and withdrawing tube feeding was reported or inferred to be longer in the following studies, ranging from within 48 h of commencing ‘fortisips’ [24], to 2–7 days from commencing free-fluids on POD4 and puree from POD5 with consideration of NGT/PEG removal from POD7 [36], 3(1.5–4.5) days [44], 3.9/5.6 days [49], 5–13 days [35] and 16 days [42]. Withdrawing EN varied from POD7 (NGT/PEG), with average time to ‘sustained’ oral intake POD8 (ERAS group) and 10 (TRAS group) [36], to POD13(5–63) (NGT) and POD141(13–636) (PEG) [35] and POD114[16–367] (PEG) [42], 7.6 ± 2 days (intervention group) and 15.9 ± 12 days (control group) [49], 9.4 (3.8) days for the early feeding groups (who commenced oral fluids within 5 days) 13.4(9.2) days for the late feeding groups (who commenced oral intake on POD6) [6] and 24 [5-270] days [31]. One study specified the time required to achieve adequate oral intake after commencing oral feeding was an average of three days (1.5 days for patients < 65years and 4.5days for patients ≥ 65years) and was an independent factor for LOS [44]. Authors indicate that commencement of oral feeding was not automatically synonymous with adequate oral intake, concluding that reducing LOS requires not only early oral feeding, but also early established oral intake. This was consistent with Højvig et al., who reported that the ability to consume sufficient nutrition orally during the postoperative period was a major limiting factor for discharge, suggesting the option of discharging with NGT or establishing a PEG, with an outpatient regimen for return to oral feeding [37]. Similarly, Stramiello et al., reported that it is unrealistic to assume that patients who begin taking oral intake early after surgery will be able to take adequate nutrition solely by mouth, recommending a gradual reduction in NGT or gastrostomy feeding, adjusted as oral intake and swallow rehabilitation improves or declines [31].

Rationale for adequate nutrition included: avoiding malnutrition, improved wound outcomes and recovery, decreased LOS, decannulation time, costs and complications. Conversely, inadequate nutrition was associated with poorer outcomes e.g. flap loss, earlier flap loss, infection and wound dehiscence [11].

There were limited studies that identified factors associated with nutritional adequacy. In two studies, overweight status was associated with a failure to reach nutritional targets, whereby calculating optimum nutrition may be substantially less than preoperative intake [11]. In addition, increased weight loss within 2 weeks of surgery (which may be associated with the increased presence of medical comorbidities (ASA ≥ 2) in these patients) [42]. In one study, more patients with nutritional inadequacy had tracheostomy and bilateral neck dissection [11] and it was inferred that older patients are more prone to inadequate oral intake [44].

Factors associated with improved nutrition delivery/adequacy included a higher number of days with oral feeding [11, 44], and an MDT approach including a specialist in dysphagia rehabilitation [44], as swallowing may aggravate pain and result in patients limiting their food intake [45]. It was also inferred that early personalised swallowing rehabilitation intervention postoperatively can reduce weight loss and improve nutritional status [48, 49] and dietitians involvement in providing calculated optimum nutrition [42].

No studies were identified that reported on patient experiences of tapering from tube to oral feeding and impact on nutritional adequacy. Healthcare professional involvement for tapering from tube to oral intake including assessment of nutritional adequacy was reported in sixteen studies. General postoperative nutritional intervention/assessment was reported as dietetic-led (ideally with experience in HNC) [41–43, 50], nurse-led [37] or in consultation with the MDT [8, 40, 50]. Specifically tapering from tube to oral intake, removal of tubes or conversion to gastrostomy was dietitian-led ± SLT (24, 33, 35) or nurse-led [45, 49] with community support for NGT discharge including occupational therapists [34, 37].

Assessment of nutritional adequacy was dietetic-led (or inferred to include dietetic assessment) [28, 42, 43] or nurse-led [11, 37, 45]. Prescriptions of EN were reported/inferred to be dietetic-led [38, 41–43, 50] or nurse driven, with one study specifying that formal nutrition prescriptions were rarely done by physicians or dietitians [11].

### Objective 3: areas for further enquiry

Areas for further enquiry are summarised in Table 2 in narrative form.

Five studies reported further prospective randomised trials/studies are required to verify findings advocating the safety of early oral feeding in order to increase confidence in the non-inferiority of early oral feeding in HNC free flaps, integrating functional outcomes to assess benefits [6, 7, 15, 31, 52] and defining/evaluating an evidence-based protocol for early oral feeding including patient reported outcome measures [33] and QOL measures [22].

One study recommended future studies should extend inclusion beyond free-flaps, to also include other reconstructive flaps (e.g. pedicled and local) which are commonly used [52].

Five studies recommended further inquiry should identify factors predictive of safe commencement of oral feeding and/or identifying which patients may benefit from an early oral feeding pathway [4, 14, 22, 30, 31], indicating that studies should encompass various age groups, cancer site, histopathology, free-flap type [14] prior chemoradiotherapy, composite resection, dysphagia and tracheostomy weaning [30]. Furthermore, these studies indicate it would be challenging to design and implement a RCT as early oral feeding as an intervention may not alter risk of developing a fistula. One study identified qualitative research to explore patient experiences, and attitudes/practices of oral feeding commencement amongst HNC surgeons/wider MDT [4]. Three studies recommended multicentre prospective or larger follow-up studies on assessing postoperative personalised swallow training and dysphagia rehabilitation [44, 48, 49].

Seven studies reported a requirement for further research in generalised HNC ERAS recommendations and/or protocols which include perioperative nutrition management and patient recovery/satisfaction, as the majority are appropriated from evidence in other surgical fields [8, 24, 32, 36, 37, 39, 41].

Three studies identified further research to identify connections for achieving adequate nutrition delivery among HNC free flaps [11], and most effective means of optimal perioperative nutrition intervention [39, 50]. Two studies reported more RCTs are needed to confirm risk factors for gastrostomy in HNC free-flaps and/or development of a decision-making algorithm [2, 35] and one study reported early identification of patients at risk of NGT dependence with an outpatient regimen for return to oral intake [37].

## Discussion

The aim of this review was to map the evidence for postoperative feeding practices and nutritional intake in patients with HNC undergoing surgery with flap reconstruction. To our knowledge, this is the first scoping review on this topic. Our results indicate that enteral feeding tubes placed were either intraoperative nasogastric tube or gastrostomy pre-, intra- or postoperatively with feeds ideally commencing within 24 h of surgery. The timing/type to oral feeding varied from as early as POD1 (sterile water ± fluids ± smooth puree ± soft diet) to POD20 (fluids progressing to soft diet). Most sources defined early oral feeding as ≤5days and delayed or traditional feeding as >5days. Commencement of oral intake was typically surgeon-led ± speech and language therapy (SLT) or healthcare professional trained in dysphagia (nurses/dentists). Thresholds for nutritional adequacy and/or withdrawing enteral tube feeding varied from 60 to 100% of estimated nutritional requirements, with postoperative underfeeding common, affecting 40%. Nutritional adequacy was mainly assessed by dietitian’s or nurses ± input from surgeons and/or SLTs.

Despite the broadscale consistency in flap surgery techniques and defect subsites across the studies, we found that timing/type of first oral intake varied with some studies favouring early oral feeding (≤ 5 days) and others favouring a delayed or traditional approach (> 5 days) which recommend a NBM period of between 6-12days, and up to 20 days. The timing of oral feeding was influenced by factors including perceived safety and benefit. Delayed/traditional practices are driven by theoretical concerns of developing a complication such as OCF, leak or wound dehiscence. Other studies [5–7, 45, 54] including two systematic reviews [14, 15], challenge the ‘arbitrary’ practice of prolonged NBM, and found that early oral feeding does not lead to increased complications (including OCF), can reduce LOS and improve swallowing rehabilitation. The majority of these studies are non-randomised single-centre cohort studies which limits confidence and generalisability in their findings and the overall safety of early oral feeding. Quality appraisal of the two systematic reviews had conflicting results, despite including the same five studies [5, 6, 22, 31, 45]. Dean et al. [14], suggested all studies reached a the threshold of high quality (scoring ≥ 7 on the Newcastle Ottawa scale) and Barlow et al. [15], suggested that of the five studies, two were are critical risk of bias [6, 31], two at moderate risk [5, 22] and one (an RCT) at low risk [45] (using the Cochrane risk of bias 1 and 2 tools). It is acknowledged amongst the studies that conducting an RCT is challenging due to the complex nature of this patient group [30, 31]. For example, patients with prior radiotherapy were excluded in the published RCT (and also the registry) [51] and also randomised according to tracheostomy presence [45]. Prior radiotherapy was a factor found to be associated with OCF in another study, where oral feeding is delayed in these patients [30] but this finding was contradicted in another study [5]. Despite evidence to suggest benefits of early oral feeding, many centres remain apprehensive to this approach. Several studies cited the role of surgeon preference, confidence and experience as important determinants for oral feeding commencement. This factor cannot be underestimated; in colorectal cancer surgery, uptake of early oral feeding is not universal despite RCT level evidence and international guidelines advocating early oral feeding in this group. An evidence-to-practice gap exists, which is also related to surgeon preference/practice [59], and recent studies have used implementation science to inform interventions to reduce this gap [60].

Choice of enteral feeding tube also varied with most studies advocating NGT and/or gastrostomy when enteral feeding was anticipated to be required for a prolonged period (≥ 4 weeks); a threshold frequently cited, originating from the UK National Institute for Health and Care Excellence nutrition support guidelines [61]. One systematic review identified predictive factors for gastrostomy in HNC pedicled or free-flap surgery [2] which included advanced tumour stage and prior radiation therapy. A scoring tool to assist decision-making for gastrostomy in this group was also identified but remains unvalidated [35]. Much of the evidence to support appropriate decision-making for prophylactic gastrostomy in HNC exists for patients undergoing primary chemoradiotherapy/radiotherapy, with tools developed that have been validated [62]. Facilitating placement of gastrostomy is likely associated with resources, as these varied between endoscopic, radiological and intraoperative placement by upper-gastrointestinal surgeons.

Processes for tapering from tube to oral feeding also varied between studies. Withdrawal of enteral tube feeding was as early as the day of commencing oral feeding. Rationales for this approach were not clearly reported amongst the studies, but included: (1) reduced pharyngeal pain due to NGT presence; hence expedited NGT removal (2) enabling discharge from hospital (as removal often coincided with discharge, in part due to lack of community support with NGTs) [34]. Anecdotally, presence of a feeding tube, particularly NGTs can prevent discharge from hospital, thereby extending LOS. It is therefore likely these tubes are removed prematurely to avoid delays in discharge. Other studies advocated gradually reducing tube feeding, adjusting to oral intake and/or as swallowing improves or declined, stating that it is unrealistic to assume that patients who begin taking oral intake early after surgery will be able to take adequate nutrition solely by mouth [31] and that an average of three days from commencing oral feeding is required to achieve a nutritionally adequate intake and withdraw enteral tube feeding [44]. This approach is consistent with feeding transition protocols developed in neurosurgery, which advocate gradual reductions in tube feeding whilst building oral intake to promote nutritional adequacy and avoid compromising nutritional status due to the early cessation of enteral tube feeding [63]. No studies were identified that explored patient experiences but input from our patient and public involvement group suggested that patients often feel rushed, with tubes removed suddenly and a lack of confidence when recommencing oral feeding which could lead to self-restricting dietary intake. This is consistent with findings in colorectal surgery [64] and a ‘trial and error’ approach to eating/drinking reported in mixed HNC surgery, where patients worked out what they felt comfortable eating [65]. Furthermore, a qualitative study across the HNC care pathway found that surgery was often experienced as a poor nutritional starting point of adjuvant radiotherapy [66], indicating that this acute post-operative period is a window of opportunity to improve nutrition care in this group. Another study found that accessing healthcare professional support and information on nutritional needs post-surgery was challenging [65], and insufficient information giving remains as a broadly unmet need amongst HNC patients/caregivers [67].

A key aim of this review was to identify adequacy of nutritional intake in patients when transitioning from tube to oral feeding following surgery with flap reconstruction. We identified very few studies that investigated this concept, and this is a gap in the literature. In this review, five studies reported on nutritional adequacy, of which one found underfeeding was common (affecting 40% of patients) which incidentally also had the lowest threshold set for adequacy (60% of estimated nutritional requirements). Methods for calculating nutritional requirements and thresholds set for adequacy varied between studies and were mainly derived from recommendations within European and American clinical nutrition guidelines in generalised cancer, surgery and critical care, which are not HNC specific. Thresholds reported were 60%, 75% and 100% of estimated nutritional requirements. Studies in non-critically ill mixed-diagnosis postoperative patients have also used a 75% threshold for nutritional adequacy [55], which has been shown to be sufficient for weight maintenance in inpatients [56]. However, caution should be applied in HNC, as it has been reported that equations used to estimate nutritional requirements underestimate needs and are insufficient to prevent weight loss [68]. Furthermore, nutritional adequacy/intake was limited to macronutrients including energy and protein. Micronutrients were not evaluated and it has been reported that vitamin D, zinc and iron are prone to deficiency immediately after surgery for oral cancer [69].

### Limitations

This review has the following limitations. Firstly, this review aimed to include qualitative studies and whilst studies that included the four concepts in the search strings (HNC, flap surgery, postoperative, feeding/nutrition) were retrieved, it is possible that some qualitative studies may have been missed, and it was not possible to adapt the search strategy to reflect this. Efforts were made to minimise this by backwards citation tracking of the two qualitative studies retrieved at full-text review [26, 70] as well as a hand search. No studies met the inclusion criteria.

Secondly, forty-five abstracts were excluded at full-text review, due to non-English language. The most common languages were Japanese and Chinese, followed by Russian, German, Spanish, Italian and French. In addition, non-English databases were not searched, which could result in selection bias and reduce the generalisability of our findings [71]. Future reviews should consider strategies to enable inclusion of non-English language studies, such as costing for professional translators into grant applications, or use of language translation technology. It was also not possible to retrieve the full-text for nine abstracts. A library loan request was made to retrieve these but were not retrieved.

Thirdly, there were several articles retrieved that reported on timing to oral feeds and/or duration of enteral feeding as part of studies that predominately focussed on broad postoperative surgical outcomes. These articles were discussed amongst the research team and as they did not provide more information beyond what the included studies reported, it was agreed these would be excluded. It is possible that excluding these articles reduced the scope of the findings. In addition, several of the articles included were more broadly focussed on ERAS. These were discussed amongst the research team and as these articles specified clearly the timing/type of oral intake and/or specifying thresholds for nutritional adequacy and withdrawing tube feeding, (often as part of publishing the ERAS protocol developed in HNC units) they were included, as they addressed key components of the research question.

Fourthly, some studies reported mixed population groups, and it was not always possible to separate these. This meant that some of the findings pertain to laryngeal/other sites, but a pragmatic approach was taken, whereby, if the majority of cases (≥ 75%) pertained to the population of interest, these were included as to avoid excluding several highly relevant articles.

Lastly, type of oral intake commenced was not always clear e.g. ‘fluids’ before day 5. Fluids could pertain to water, clear fluids or free-fluids, which have implications for nutritional intake and tapering off tubes, as water has no provision of energy/protein whereas free-fluids could include nutritionally complete oral nutritional supplements. Limited use of the International Dysphagia Diet Standardisation Initiative (IDDSI) framework was noted across studies, which could assist with clarity and consistency in reporting.

### Future work

Future prospective research should verify findings advocating the safety of early oral feeding. Whilst an RCT randomising patients to early or delayed oral feeding may provide the best quality evidence and increase confidence in its safety, this study design may be challenging to design and conduct due to the complexity of the patient group and variation in practices amongst clinicians. Prospective studies that identify factors associated with safe commencement of oral feeding, choice of feeding tube and nutritional adequacy (including micronutrients) when tapering from tube to oral feeding, may be more appropriate and could identify the most suitable candidates for an early oral feeding pathway and patients who may benefit from prolonged enteral tube feeding, even once oral feeding has initiated. This review identified variation in practices for timing/type of oral feeding and enteral feeding tube placement/removal, which were likely associated with surgeon preference, unit culture/practice and resources. This could be captured in a survey and though qualitative exploration informed by behavioural science theories and frameworks amongst key HCPs involved in postoperative feeding management including surgeons, dietitians, SLT and nurses. A gap also exists exploring patient experiences of postoperative feeding practices, to understand barriers and facilitators for commencing oral feeding and achieving nutritional adequacy when tapering from tube to oral feeding and in the acute postoperative period. Very few studies have studied nutritional adequacy in the postoperative period. Future research should identify connections for achieving adequate nutrition delivery among HNC free flaps.

## Conclusion

Postoperative feeding practices in HNC flap surgery vary with timeframes for commencing oral feeding between one to twenty days, predominately led by the surgeons and/or SLT and dietitians. Withdrawing enteral tube feeding was as early as the day of oral feeding commencement to 141days. An average of three days was required to reach adequate oral intake once oral feeding was initiated. Underfeeding is common in this patient group, and thresholds used to determine nutritional adequacy were set at 60 to 100% of estimated nutritional requirements, with differing methods used to measure estimated nutritional requirements. Key healthcare professionals determining nutritional adequacy were mainly dietitians or nurses.

## Supplementary Information

Supplementary Material 1.

Supplementary Material 2.

Supplementary Material 3.

## Data Availability

No datasets were generated or analysed during the current study.
