# Supplementary material for: Postoperative feeding practices and nutritional intake in patients with head and neck cancer undergoing surgery with flap tissue transfer reconstruction: a scoping review
Source: BMC Cancer. 2026 Apr 16;26:733. doi: 10.1186/s12885-026-15973-9 (PMC13248424; doi:10.1186/s12885-026-15973-9)
Supplement: Supplementary file 3 — Supplementary Material 3. [file 12885_2026_15973_MOESM3_ESM.docx]

**Supplementary File/Table 3: Overview of studies in relation to scoping review objectives and inclusion criteria**

| **Study reference** | **Objective 1a) The timing and type of dietary textures patients are transitioned onto** | **Objective 1b) The type of enteral feeding tubes placed and duration of enteral feeding** | **Objective 2) How patients are transitioned from tube to oral feeding and adequacy of nutritional intake when transitioning in the acute postoperative phase** | **Patient or HCP experiences or involvement in postoperative feeding** | **Complications or benefits associated with a postoperative feeding practice** |
| --- | --- | --- | --- | --- | --- |
| Barlow et al (2024), Early feeding after free flap reconstruction of the oral cavity: A systematic review and meta-analysis. | Early feeding defined as:  -on or before POD5  -before POD5  -POD1 after procedure  Late feeding defined as:  -after POD5  -POD6 after 5-day NBM period  -Dependent on success of oral intake trial. | - | - | Limitation section indicates the importance of physician judgement. | Early oral intake prior to POD5 is not associated with increase in OCF but is associated with decreased LOS. No significant differences between early or delayed feeding groups for flap failure, haematoma formation, wound dehiscence, local or systematic infection, and total complications. |
| Dort et al (2017), Optimal perioperative care in major head and neck cancer surgery with free flap reconstruction. A consensus review and recommendations from the Enhanced Recovery After Surgery Society. | Oral diet first choice for tolerating patients but due to large variation in functional deficit, some patients able to resume oral intake whilst others require NBM with EN.  Limited number of studies assessing EOF therefore application should be evaluated on an individual basis. | Recommendations:  -Use of a standard polymeric formulae  -Insufficient evidence for immunonutrition.  -Oral diet first choice for tolerating patients otherwise tube feeding within 24hrs -Nasoenteric tube or gastrostomy (where adjuvant therapy /prolonged EN is anticipated at diagnosis). PN rare but indicated in the absence of normal gut function/ when enteral access contraindicated. | Recommends that nutritional interventions should be individualised according to nutritional status and surgical procedure. | Recommends that nutritional interventions should be in consultation with the MDT.  Optimal timing of reintroduction of oral intake should be individualised in consultation with the surgical team, dietitian and speech pathologist. | Some benefits of immunonutrition but inadequate evidence to justify routine use. |
| Nurkkala et al (2021). Causes of nutrition deficit during the immediate postoperative period after free flap surgery for cancer of the head and neck. | First day of oral feeding wad 5[3-7] for adequate group and 6[5-8] for low group.  Days of oral food intake (within 10-day postop study period) was significantly more for the adequate group (6[4-8] days) than low group (5[3-6] days).  Discussion mentions that problems in surgical area such as swelling, pain, poor tissue healing and attending surgeons’ customs may postpone oral feeding initiation. Impaired food intake after flap surgery may result from dysphagia, odynophagia, anatomical and physiological reasons. | Patients administered IV 5% dextrose, PN and EN from (POD1). Type of tube not specified or when this was placed. | Dietary intake (food) was estimated by intake in (ml) based on average hospital diet of 1800kcal/day.  Energy requirements estimated individually using ESPEN/ENT-UK guidelines (30kcal/kg/day, IBW) for surgical patients. 60% threshold of energy requirements to mark nutritional adequacy (n=131/60% achieved this). A higher IBW was associated with not reaching nutritional targets whilst higher number of days with oral feeding associated with a decreased odds ratio for impaired nutrition delivery. More patients in the inadequate group had tracheostomy and bilateral neck dissection. | Nurse driven nutrition support. Formal prescriptions were rarely done in this study by the attending physicians or dietitian.  Initial judgement of oral food delivery is ultimately completed by the attending surgeons’ evaluation and customs, which may postpone oral feeding initiation. | Sufficient nutrition after free flap reconstructive surgery is beneficial and early oral food intake is associated with increased nutrition delivery.  No difference in flap loss but inadequate group lost flaps earlier and had more postoperative infections. |
| Coyle et al (2016). Enhanced recovery after surgery (ERAS) for head and neck oncology patients. | Protocol stipulates SLT assessment from POD2 and if safe for water, otherwise SLT review for 'fluids' POD3/4/5/6/7-10 for fluids if appropriate with aim for 'some oral intake' by discharge.  States patients with PEG will generally use this until safe to resume an oral diet. For patients where PEG is not deemed necessary, scarcity of evidence for optimal timing of oral feeding after surgery. | Protocol stipulates EN commenced via PEG or NGT within 12hrs of surgery (with 90% in the compliance study given postoperative EN within 24hrs).  Decision made for gastrostomy at pre-operative MDT appointment and placed in theatre. | Protocol stipulates continued EN whilst commencing fluids as required POD2-10.  On discharge aim for NGT tube to be removed, adequate nutrition intake, some oral intake and/or patient able to care for tubes (gastrostomy education from POD4)  Dietitian review POD2/3 and POD4. SLT review from POD2 and commence water if safe in addition to NGT/PEG feeding. | ERAS in HNC requires an MDT. Protocol developed by OMFS and ORL surgeons, anaesthetists, dietitians, physiotherapists, SLT (specifies for assessment of fluids) and nurses.  Inferred that SLTs hep guide safety of oral feeding and dietitians guide nutritional assessments.  Patient participation encouraged via a diary and checklist, which guides what they can expect each day. | LOS reduced since introducing the ERAS programme in HNC.  Authors state that the reluctance to commence oral feeding early includes concerns about the safety of swallowing, risk of aspiration and fistula formation (around the reconstructed intra-oral site and subsequent leakage into the neck) |
| Stewart et al (2023). Predictors of gastrostomy tube placement in patients with head and neck cancer undergoing resection and flap-based reconstruction: systematic review and meta-analysis. | - | Factors not associated with increased risk of gastrostomy:  -Gender, resection type, defect site and laterality of ND on univariable analysis.  Factors associated with increased risk of gastrostomy:  -Cancer stage IV/ recurrence compared to stage III.  -Prior RT compared to postoperative RT.  -Among free flaps, RFFF less likely compared to RA and LD. No differences between pedicled and free flaps.  -On univariable analysis: age >70 years, preop BMI <18.5, intraoperative tracheostomy, oropharyngeal versus segmental mandible tumour resection and >50% tongue resection compared to FOM resection. | - | Conclusion states MDT approach but specific HCPs not mentioned. | Factors identified can support appropriate patient selection for gastrostomy tube as a route of EN, which challenging in this patient group. |
| Guidera et al (2013). Early oral intake after reconstruction with free flap for cancer of the oral cavity. | Patients divided into early oral feeding (fluids orally by POD5) and later oral feeding group (began oral feeding from POD6). LOS was significantly shorter for the early group 11.9(3.6) days than late group 18(9.4) days. | Patients likely to require prolonged nutritional support, such as postop RT to the primary site and neck bilaterally routinely have a PEG inserted pre-op or in the immediately post-op (n=9). Remaining had a NGT placed at induction or immediately after the tracheostomy, before the operation.  NGT duration 9.4 ± 3.8 days and 13.4 ± 9.2) days (early and late groups). | Postoperative weight change measured (at discharge) with weight loss -1(3.8)% (early group) and 2.6(4.1)% (late group) | Decision to introduce oral intake depended on individual surgeon preferences. One surgeon routinely started early feeding once the patient could dispose of saliva. Other surgeons prescribed late oral feeding. | EOF associated with significantly shorter LOS and does not result in increased postoperative complications including failed or partially failed flaps, repeat operations, OCF.  NGT and tracheostomy intubation time, age, number of complications and % weight change was not significantly different between the two groups. |
| Navarro Vila et al (1989). Enteral nutrition in patients with tumours of the head and neck. | Doesn't state in results but infers from introduction that a 7-day NBM period is necessitated to avoid contamination, infection or dehiscence of the wound. | Fluid electrolyte replacement therapy given first 48hours post operatively followed by NGT feeding (pump infusion, 1.0kcal/ml feed). Only patients who were stable and tolerated EN progressed to a faster feed rate and night rest of 8hrs.  Doesn't state when NGT placed. Duration of NGT feeding at least 7 days. | Energy intake estimated using Harris Benedict formula (BMR x 1.3 activity factor and 1.4 stress factor). Average daily energy requirement was 2090 ± 270kcal/day. 50% estimated calories given on first day, increasing to 100% requirements on the second/following days. When EN was suspended, a new nutritional evaluation was made. Patients lost an average of 1.2-1.8kg at the end of EN (not significant). | - | Administration of EN avoids protein energy malnutrition in the postoperative period and infection by food remaining in the oral cavity.  Complications with EN included diarrhoea (n=8) which settled when feed was stopped and restarted at a slower rate (or Lomotil used in severe cases), pneumonia (n=1) following aspiration of gastric juice and hyperglycaemia (n=1 diabetic patient). |
| Dawson et al (2017). Factors affecting orocutaneous fistula formation following head and neck reconstructive surgery. | The day oral intake started, and formation of fistula was not statistically significant, but 8/11 of patients who developed a fistula started oral intake before day 8. Following these results, unit practice is that patients with prior CRT do not commence oral intake until at least the 8th postoperative day. | Gastrostomy or nasogastric tubes (time of placement not specified).  Duration of feeding reported in the n=11 who had a fistula:  -n=2 with pervasive fistula underwent 60-day NBM and EN via NGT or gastrostomy  -n=9 restarted oral intake after 5-day NBM from the point the fistula was identified to facilitate optimal healing. | - | Restarting oral intake is decided on an individual basis with collaboration between the surgeons and SLTs (no protocol exists). MDT should be overt at the outset about the risk of fistula in patients with prior CRT.  Discussion references that the main priorities of patients with HNC include being able to swallow all food and drinks. | The day oral intake started and formation of fistula was not statistically significant, but 8/11 who developed a fistula started oral intake before day 8. Some fistulas do not appear until 24hrs after oral intake has restarted, so high-risk patients should be monitored carefully. No surgical interventions were used to aid healing of fistulas.  All possible causal associations between surgery and fistula was analysed including tumour site/surgical details, only significant association was previous CRT. |
| Stramiello et al (2021). Timing of postoperative oral feeding after head and neck mucosal free flap reconstruction. | Cases were divided into early (EF n=24 ≤POD5) and late oral feeding (LF n=80 >POD5). Timing of conservative oral intake ranges 10 to 20 days, an exact definition of early oral feeding has not been recognised so followed Guidera et al 2013 convention.  Timing of oral feeding depended on practising surgeons, tissue quality, prior radiation and/or other treatments, comorbidities, baseline swallow function, and reconstructive characteristics.  Factors supporting early oral feeding: -Surgery as initial oncologic treatment  -Oral cavity or smaller primary tumours.  -EF group were all oral cavity and mandible reconstruction, and less likely to have prior RT.  Factors supporting later oral feeding include:  -Prior radiation  -Poor baseline swallow  -Comorbidities (hypothyroidism and diabetes, associated with poor wound healing).  -Age, defect size and proximity of a suture line to pooled saliva were not critical factors. | NGT or gastrostomy (time of placement not specified)  Duration: EF patients had feeding tubes for median postoperative duration of 24 days (range POD5 to 270).  Once a patient is able to maintain nutrition on oral feeding alone, the enteral feeding tube can be withdrawn. | Majority of late feeding patients required more time to reach their penultimate swallowing function score than early feeding patients and early feeding groups who progressed to full oral diet earlier (by POD30 but this was not significantly different by POD60/90).  Discussion states that postoperative swallow function will gradually improve over weeks or months depending on extent of operation and previous treatments and that it is unrealistic to assume that patients who begin taking oral intake early after surgery will be able to take adequate nutrition all by mouth. Gradual reduction in tube feeding should adjust as oral intake and swallow rehabilitation improves (or declines with adjuvant therapy). Once a patient can maintain nutrition on oral feeding alone, the enteral feeding tube can be withdrawn. | Timing of PO institution depended on practicing surgeons experience and confidence with attaining a watertight flap inset. | EF may improve swallow functional outcomes earlier.  Fistula incidence within the first 3 months of surgery was 0% early and 16.5% in late groups and 9/13 had prior radiation. Fistula diagnosis ranged from POD1 to 43 in previously irradiated patients and POD5 to 21 in those not previously irradiated. The patient with a fistula on POD1 was treated for mandibular ORN + free flap.  In properly selected patients, EF may not increase fistula risk. EF is associated with expedited diet advancement contributing to better patient QOL. This study does not support that delaying oral feeding will prevent a fistula. |
| Imai et al (2024). Enhanced recovery pathways for head and neck surgery with free tissue transfer reconstruction. | Considering limited evidence, early postoperative oral intake should be carefully assessed case-by-case and may be difficult due to decreased swallowing function after surgery. Surgeons must also assess risk of postoperative fistula development. | Early EN described as being 'oral or tube feeding that begins within 24hrs postoperatively, or at the latest, 48hours postoperatively'. In high-risk patients, e.g. free jejunal reconstruction, early EN with low-speed continuous administration reduces risks of enteral feeding intolerance.  Perioperative immunonutrition has no definitive evidence in head and neck surgery. | - | MDT as part of ERAS - head and neck /plastic surgeons, nurses, gastroenterologists, anaesthesiologists, dentists, dietitians, dental hygienists, speech therapists, pharmacists, physiotherapists and social workers.  Surgeon considers risk of fistula with early feeding. SLT assess swallowing function. Dietitian explains early EN and swallowing adjusted meals. | Early EN reduced LOS and complications (references appropriated from non HNC sources). When considering the route of feeding (oral or enteral), surgeons must consider the potential risk of fistula, ‘especially when early feeding is given from the more cranial side’ (no further explanation of what this specifically means). |
| Brady et al (2021). Early postoperative feeding: an investigation of early functional outcomes for oral cancer patients treated with surgical resection and free flap reconstruction. | Conservative approach described per Kerawala *et al*., 2021 (NBM for up to 12 days following surgery). Early feeding protocol stipulates on POD1: sips water ± smooth puree, working with dietitian to inform non-oral feeding plan.  Results: 100% reviewed for bedside swallowing evaluation on POD1 and the majority were able to tolerate oral fluids on POD1 (86%) and continued to progress to a smooth puree (IDDSI4) and soft and moist diet (IDDSI 5). | NGT and gastrostomy. Protocol stipulates consideration of gastrostomy preop or postop (POD1 and 7-14).  Results state median time to NGT removal was 7[1-9) for n=14. Abstract states time to NGT removal was 6[3-15] days (unclear if this was 100% of the cohort).  41% had gastrostomy; preop (17%), due to poor baseline swallowing function and postop (24%). Decision for gastrostomy was multifactorial including baseline swallowing function, patient preference, close working with dietetics and potential need for adjuvant treatment (62% required post of RT/CRT). | Early feeding protocol stipulates on POD1: sips water ± smooth puree, close working with dietitian to inform non-oral feeding plan. POD3-5: sips water ± smooth puree, ± soft moist diet (IDDSI5), ± soft and bite sized diet (IDDSI6), close working with dietitian to inform non-oral feeding plan ? removal NGT. If any concerns re swallowing safety, instrumental swallowing evaluation (FEES/VFS). POD7-14: sips water ± smooth puree, ± soft moist diet (IDDSI5), ± soft and bite sized diet (IDDSI6) ± regular easy-chew diet (IDDSI7), close working with dietitian to inform non-oral feeding plan. ? removal NGT or conversion to gastrostomy, as per VFS/FEES if indicated. | SLT: for clinical and instrumental evaluation of swallowing.  Dietetics and SLT: Decision making for gastrostomy and tapering from tube to oral intake.  Dietetics: for recommending removal of NGT/conversion to gastrostomy. | 7% remained on fluids only at discharge and 3% NBM due to persistent OCF or total flap failure.  Transient OCF was observed in 1 patient at POD17 which resolved with antibiotics and 7-day NBM period (then resumed soft and moist diet (IDDSI 5). 2 other patients had a OCF (POD 1 and 3) but had not yet commenced oral intake/been reviewed by SLT prior to discovery of the fistula. 3% had flap loss. Patients who had OCF were T3/4, N2B/2C and required tracheostomy.  Early oral feeding protocols have potential for shorter LOS (10 compared to 21 days in historical cohorts) and earlier swallowing rehabilitation. |
| Cook et al (2022). Oral feeding commencement following free or pedicled flap reconstruction for oral cancer: Experience from a tertiary referral centre. | Oral feeding commenced after 11.9 ± 4.1 days. In free flaps (n=24), 12.3 ± 3.9 days and pedicled flaps (n=10) 10.9 [4-20] days, no significant difference. Discussion states this is consistent with 'late feeding' defined as >5-7 days or conservative management of 6–12-day NBM. Only 1 patient commenced oral diet/fluids under 5 days, majority 7 to 12 days. Per defect: FOM 14[14-14] days, Maxillectomy 10[9-15] days, Mandibulectomy 9[9-16] days, Rim resection 10[4-20] days, Partial glossectomy 10[7-22] days, hemi glossectomy 14[7-17] days, and Buccal 10[10-10] days. | 85.3% (n=29) NGT and 14.7% (n=5) gastrostomy (PEG). Centre has a prophylactic gastrostomy service, for when it is anticipated surgery will lead to long-term feeding and/or adjuvant RT required.  Duration of tube feeding 12.9 ± 5.3 days overall. In free flaps (n=24) 13.8 ± 5.2 days and in pedicled flaps 10.9 ± 5.4 days (no significant difference). | - | - | Weak positive correlation reported between earlier initiation of oral intake with shorter LOS.  Clavein dindo complication scores included but not specific to relationship with oral feeding. |
| Denholm et al (2022). Factors determining postoperative length of stay and time to resumption of feeding following free flap reconstruction for oral cancer. | Most patients resumed ‘sips’ by 5 days postop. 84% recommenced sips whilst inpatients, n=64 progressed to free fluids, n=58 resumed a puree diet and n=4 resumed a ‘normal’ diet.  Patients with a tracheostomy were less likely to resume sips and female gender was significantly associated with a longer time to pureed diet. Age and tracheostomy were significantly associated with reduced likelihood of resuming free fluids. ICU stay of ≥2 days was associated with a longer time to resumption of free fluids. Some evidence that bone resections reduced the likelihood of resuming a normal diet, but this was not statistically significant on multivariate analysis. | NGT or gastrostomy (PEG). n=66 (66%) of patients were discharged from hospital with an NGT. | Outcomes for time to resumption of each level of feeding, were with a binary outcome of whether a patient was discharged with/without NGT.  Authors state that community support is integral in discharging patients home with NGT, and the reason this service is available in their area may explain the lack of association between overall LOS and time to resumption of feeding. | Community support integral for discharging with NGT. | Overall LOS 14 [7-48] days was not significantly associated with time to resumption of feeding.  Authors comment that the longer ICU stay with resuming free fluids may be due to the decisions to keep patients NBM as a precaution following complications, or unplanned return to theatre. |
| Eley et al (2012). A review of postoperative feeding in patients undergoing resection and reconstruction for oral malignancy and presentation of a pre-operative scoring system. | - | PEG indicated when nutrition support was required ≥4 weeks. Consideration to tumour volume/site, adjuvant RT.  PEGs are routinely inserted in theatres after airway secured pre-op. Inserted in n=120 (n=5 inserted in endoscopy, 14 (2-26) days before operation and n=115 at the time of surgery, with 1 jejunostomy. Mean duration 795 (310-1344) days. NGTs end of the operation (n=24 duration was 13[5-63] days of which 3 had a PEG at 21 and 30 days).  Scoring system developed: total score 0-3 indicated NGT and ≥4 PEG. Generated from: Score 4 for: total glossectomy.  Score 3 for: ASA grade III-IV, T3/4 stage, alcohol >40 units/week, or ≥ 2 simultaneous free flaps. Score 2 for: fibula/ ALT/ LD flap, adjuvant RT > one field, excision of 1/3 tongue, tonsilla fossa/pharyngeal/soft palate, mandibulectomy, and medications >3. | Discussion indicates that there may be concerns about use of PEG in delaying a return to normal feeding due to protracted disuse of swallowing muscles for deglutition.  n=77 had PEGs removed, mean duration of use from insertion to request from removal 141(13-636) days and date to actual removal 212(27-733) days. n=9 used PEG for <28 days, for which NGT may have been more appropriate; all had minimal coexisting medical problems with ASA grade I or II and were treated with partial glossectomy and RFFF.  Discussion suggests that to reduce the time to PEG removal after request (mean 71 days), cut and push PEG removal could be explored. | Decision to remove the PEG is multifactorial, and patients are assessed and followed up by the dietitian and speech and language therapy teams.  Gastrostomies routinely placed by gastroenterologist in theatres after airway secured pre-op. | Scoring system can assist clinicians to select correct tubes in 92% of patients. Identifying gastrostomy can avoid NGT and associated complications (naso irritation, mucosal ulceration and displacement).  Patients required a mean of 1.9 NGT (1-5) tubes per patient and 2 developed complications of their NGT (aspiration pneumonia).  NGT for > 28 days (33 and 63 days) in n=2 due to 1) perforated colonic diverticulum 2) return to theatre - respiratory failure.  PEGs: n=1 had diabetic gastroparesis and required TPN. n=10 needed imaging after PEG due to abdo pain, early sepsis, confusion of concerns of a leak (average 6.3 days post insertion, range 3-12 days). Leak in n=1, n=3 abdo issue and n=5 normal N=19 developed minor complications (16%). n=4 abdo pain, n=3 feed, n=2 leakage around stoma, n=7 overgranulation, n=2 infection around skin site, n=1 accidental removal. |
| Bater et al (2017). Enhanced recovery in patient having free tissue transfer for head and neck cancer: does it make a difference. | ERAS programme developed stipulates on  POD4: starts sips of water building up to free fluids if safe to swallow.  POD5: start puree diet.  POD6: continue puree diet. | ERAS programme developed stipulates: POD1: enteral feeding starts (NGT or PEG).  States patients receive nutritional assessment preoperatively which enables early placement of a PEG in malnourished patients at a weekly endoscopy session that is ringfenced for HNC. | ERAS programme developed stipulates: POD1: enteral feeding starts (via NGT or PEG). POD2: SLT assessment If no tracheostomy. Increase rate of enteral feed. POD3: daily SLT review, consider decannulation of tracheostomy cuff. POD4: consider decannulation if tolerates cuff down for 24hrs, starts sips of water building up to free fluids if safe to swallow. POD5: start puree diet. POD6: continue puree diet. POD7: remove NGT/PEG and plan for discharge. POD8: discharge.  Average time to ‘sustained’ oral intake (not defined) was 10 days for TRAS and 8 days for ERAS group. | Multidisciplinary ERAS programme; OMFS surgeons, anaesthetists with interest in HNC, CNS.  Before feeding restarted, all patients are assessed by SLT to check swallow safety. | LOS and time to mobilisation significantly shorter in ERAS group which has cost savings without increased in complications (including OCF) or readmissions.  No patients in either group developed an OCF as a result of early feeding. |
| Højvig et al (2022). Enhanced recovery after microvascular reconstruction in head and neck cancer - A prospective study. | States in methods ERAS protocol stipulates landmarks in terms of time to oral feeding but doesn’t specify what these are. | NGT placed in 90% ERAS and 96% TRAS groups. Conversion to PEG in 7% ERAS and 12% TRAS groups.  NGT duration significantly shorter in ERAS than TRAS groups: 13.3[1-30] and 22.7[1-126] days.  Discussion states that early identification of risks of NGT dependency could prevent prolonged hospitalisation and allow discharge with NGT or PEG. | Discharge with NGT or establishment of PEG could enable discharge of >80% by POD7.  NGT tube duration was synonymous with removal and sufficient oral intake which was shorter in ERAS than TRAS group.  Discharge criteria in ERAS protocol stipulates sufficient/adequate nutrition intake (of calculated requirements) and monitored daily.  Discussion states that the ability to consume sufficient nutrition orally postoperatively limited discharge and problems with wound healing, re-operations and complications at the recipient site can delay the time to oral nutritional intake and some may be dependent on tube feeding. An outpatient regimen for resuming oral feeding is important for convalescence and QOL. | Unavailability of occupational therapists cited as an issue related to prolonged LOS due to a nutritional problem.  Discharge criteria (which includes nutritional adequacy) in ERAS programme monitored daily by nursing staff. | ERAS programme associated with significantly shorter LOS without increasing the risk of infection, surgical site complications or readmission. Discharge with NGT or establishment of PEG could enable discharge of > 80% of patients by POD7.  10% had prolonged LOS due to nutritional problems: 7% due to unavailability of occupational therapist and 3% due to lack of compliance (one was discharged with NGT and one had a PEG placed) |
| Hwang et al (2023). Intraoperative enteral nutrition feeding in free-flap healing after reconstruction surgery for head and neck cancers. | - | NGTs inserted by otolaryngologist after tumour resection and before commencing free flap.  EN started intraop with a feed comprising 100kcal/100mL, 4.2gprotein/100mL, 3.8g fat/100mL prescribed at 10-20mL/hr (mean volume 134 (20-350) mL) depending on the residual gastric content aspirated and continued postop. The fasting group started feed postoperatively. Average operation duration was 12.3 ± 2.7 and 13 ± 2.8hrs, fasting and feeding groups. Average fasting time was significantly shorter in the feeding group 20.2 ± 4.5hrs than the fasting group 38.2 ± 5.8 hrs. | - | Surgeons placed NGT during operation.  Dietitian prescribed rate/volume of enteral feeding. | Perioperative fasting time shortened due to intraoperative NGT feeding with EN. Intraoperative EN significantly reduced the occurrence of flap wound dehiscence and edge necrosis and LOS.  Partial and total flap failure similar between the two groups (14.2%). Rate of dehiscence or edge necrosis was significantly lower in the feeding group: n=6 vs 0 for fasting versus feeding. LOS shorter and hand grip strength better preserved in the feeding group. Plasma concentrations of interleukin (IL)-6 and IL-8 increased (significantly) more in the fasting group. Perioperative adverse events did not differ. Pulmonary aspiration in anaesthetised patients is a major concern and predefined patients who were not at increased risk of regurgitation or bowel obstruction did not result in pulmonary aspiration. |
| 1 reference with 2 reports: 1) Dean et al (2024). Early oral feeding and its impact on postoperative outcomes in head and neck cancer surgery: a meta-analysis 2) Dean et al (2024). Does the timing of oral feeding affect the fistulisation risk among head and neck cancer patients undergoing free flap reconstruction? | Introduction states traditional practices are NBM for 6-12 days (citing Skoner *et al.,* 2003 and Sinha *et al.,* 2004).  Early oral feeding defined as ≤ 5 days or earlier postoperatively (using Guidera *et al.,* 2013). Patients who received oral feeding >5 days postoperatively were assigned to the late feeding group. Patients started on fluids and progressed to a soft diet. | - | - | Infers in conclusion that surgeons are responsible for implementing early oral feeding protocols in HNC free flap surgery. | Early feeding significantly decreased LOS compared to late feeding and did not increase the risk of complications (no difference between early and late groups for fistulas, strictures, haematomas /seromas, dehiscence and flap failure).  No differences observed between early and late feeding groups amongst patients who had received preoperative chemo/radiotherapy. |
| Kinzinger & Bewley (2017). Perioperative care of head and neck free flap patients. | States common practice of delaying oral intake stems from concern for wound dehiscence, fistula and aspiration. References Guidera *et al.,* 2013 in support of early oral feeding and that delaying oral feeding >6 days does not decrease complications. States oral feeding is commonly resumed 5-7 days postoperatively. | Review supports early initiation of enteral nutrition and states enteral feeds are started on POD1 in head and neck free flap patients (references Dort *et al.,* 2017 and Coyle *et al.,* 2016) and at the authors institution, a university hospital, in the USA. |  | States decision for resuming oral intake lies with the operating surgeon’s judgement. | References postoperative nutrition can improve wound outcomes, decrease LOS and costs and early oral feeding is associated with diminished LOS.  References studies that indicate no increases in complications (OCF or flap failure) with early oral feeding. |
| Vincent et al (2019). Perioperative care of free flap patients. | States that surgeons are often wary of restarting an oral diet soon after head and neck free flap reconstruction, especially when this involves the oral cavity and pharynx. This is due to theoretical risk that early oral intake could compromise new suture lines and increase the risk of fistula formation.  References that studies suggest early oral feeding (defined as <6 days) does not increase OCF formation nor flap relayed complications, whilst reducing LOS. These studies clearly recommend and demonstrate the importance of postoperative nutritional support. | States that an oral diet provides the best nutrition and should be first choice for patients tolerating otherwise enteral feeding should be initiated within 24hrs after surgery.  States placement of a gastrostomy tube can be considered both pre- and post-operatively when a prolonged nutritional support is anticipated or when expected adjuvant therapies may worsen or cause dysphagia. References evidence for immunonutrition varies with mixed results and current evidence supports standard enteric nutritional support. | References that adequate nutrition before and after head and neck flap surgery improves outcomes. States adequate nutrition is important to maintain postoperative needs in addition to leading up to surgery. States that nutritional interventions should be individualised to each patient. | Infers that surgeons are the decision makers for restarting oral diet after free flaps.  States that nutritional intervention is best developed in consultation with the multidisciplinary team but does not specify what professionals this includes. | References that studies suggest early oral feeding is advantageous by reducing LOS. Wariness of early oral feeding is due to theoretical risk that this could increase the risk of fistula formation. |
| List et al (2022). Enhanced recovery after surgery, current, and future considerations in head and neck cancer. | References Kerawala *et al.,* 2021 findings of early oral feeding on POD1 after free flap is feasible. References Stramiello *et al.,* 2021 findings of early oral feeding ≤ POD5 in properly selected patients. | Early enteral feeding within the first 24hrs is advocated (in this review high level recommendation with conditional level of evidence). References the ERAS programme from Coyle *et al.,* 2016 which advocates enteral tube feeding within 12hrs of surgery. | High level recommendation with strong evidence to support that postoperative nutrition plans should be made by a dietitian who has experience with HNC patients. | Broadly mentions the role of an MDT for ERAS.  States dietitians with experience in HNC should be involved in managing patients malnourished or at risk and postoperative nutrition plans (High level recommendation, strong evidence). | References studies that report that early oral feeding is associated with reduced LOS (by>7 days), may expedite swallow recovery and is not associated with increase fistula. Early enteral feeding within the first 24hrs is advocated by many guidelines as it reduces septic and major medical complications. |
| McAuley et al (2015). Early feeding after free flap reconstruction for oral cancer. | Introduction states there early oral feeding is thought to increase the risk of OCF, and surgeons have traditionally adopted a conservative approach of 6-12 days NBM, as early use of the tongue and pharynx place stress across the intraoral closure, leading to flap dehiscence and may contaminate the neck and lead to OCF.  Sterile water: POD1 (50%) POD2 (50%). Average 1.5±0.5 days.  Puree or sloppy diet: POD3 (50%) POD4 (30%) POD5 (10%), POD7 (10%). Average 3.9 ± 1.3 days. | States NGT but does not specify when these were placed / by whom. | States that by POD4, 80% could tolerate a puree diet at which point the NGT was removed. n=1 could tolerate by POD5 and the other by POD7 (who had a mandibulotomy to help facilitate resection of the FOM tumour as well as ND and RFFF reconstruction).  States oral intake was monitored closely to ensure adequate calorific intake. Does not state how this was measured or estimated and who by. | Infers in the introduction that surgeons are decision makers for oral feeding commencement. | Early oral feeding is associated with shorter hospital stay, which was 11[5-16] days. Main reason for LOS>7 days were dressings and social care.  Conservative feeding views may originate from general surgery, but ideas have changed due to economic benefits of shorter LOS.  No failed flaps, development of OCF or neck complications. |
| Niziol et al (2024). A rapid recovery protocol (RRP) for head and neck oncology patients undergoing resection, free flap reconstruction and tracheostomy: a feasibility study. | RRP protocol stipulates:  POD3: SLT assessment for sips of water and plan for next 48 hrs. Decannulate if appropriate.  POD4: start ‘fortisips’ if appropriate and after surgical review to rule out dehiscence. | RRP protocol stipulates a CXR for NG positioning ordered post-op (day of surgery) therefore inferred intraoperative placement/ use of NGT the day of surgery. | RRP protocol stipulates:  On POD4: to start ‘Fortisips’ (if appropriate after surgical review, and oral nutritional supplement drink, equivalent to free fluids), for dietitian review with a plan for next 48 hrs and NGT to be removed if taking 75% of recommended calories. Does not state how recommended calories were calculated/by who.  On POD5: discharge Between POD8-12L: to review in rapid discharge clinic.  At 6 month follow up, all patients were orally fed. | Introduction states ERAS aims to empower the patient and reduce trauma of surgery.  A multidisciplinary approach designed the protocol which included surgeons, anaesthetists, nurses, intensivists, physiotherapists, SLT and dietitians.  States the surgical team, SLT and dietitian would review the patient regarding building up oral nutrition when appropriate. SLT assessment for sips water, surgeon for fortisips and dietitian involvement during transition. | RRP group were decannulated 5 days earlier and discharged 7 days earlier compared to the matched control group, amounting to cost savings of £9,955/patient, £15,9280 total.  States patients receive a surgical review to rule out dehiscence prior to commencement of ‘fortisips’ (which is an oral nutritional supplement drink, would be equivalent to free fluids). |
| Chandu et al (2003). Percutaenous endoscopic gastrostomy in patients undergoing resection for oral tumours: A retrospective review of complications and outcomes. | - | Introduction states NGT used to aid nutrition in patients undergoing surgery but PEG now advocated as an alternative.  PEGs placed by upper gastro-intestinal surgeons mostly intra (after induction of general anaesthesia) or preoperatively, as a day procedure under light sedation and local anaesthesia. 41 PEGs inserted at the time of resection, 7 placed 6.2 (1-17) days before surgery and 2 had PEGs postoperatively.  Feeding planned to start on POD1. | PEGs duration n=31 was 114 [16-367] days. n=10 died (not related to PEG) with duration 470[28-2136] days. n=7 still using PEG at time of study, duration 413[70-913] days.  Mean (statistically significant) decrease of 2kg/2.95 weight from preoperative weight to 2 weeks postoperatively - authors discuss is likely due to metabolic response to trauma. Over next 4-8 weeks further weight loss of 1kg (not significant), giving total weight loss of 4.7% of preoperative weight. Trend that overweight patients lost more weight. Discussion states findings indicate patients are likely adequately nourished in the immediate postoperative period with the PEG, 80% had medical comorbidities that may affect nutritional status and the ability to convalesce. PEG can be retained longer if having radiotherapy. | States dietitians are involved in calculating optimal nutrition and that the reason overweight patients lost more weight may represent the dietitians involvement in providing calculated optimum nutrition, and it is probable this was substantially less than preoperative intake.  States as part of a team approach, PEGs were placed by upper gastrointestinal surgeons (with the majority placed by the senior surgeon), with 1 placed radiologically where the scope could not be passed. | Benefits reported of PEG placement in oral cancer (all patients undergoing major resection of oral tumours in this unit have a PEG placed for nutritional support), preferably at the time of resection.  Authors discuss that intraoperative placement allows immediate or early enteral nutrition after surgery, avoids an extra procedure, and postoperative immobility aids healing of the PEG site and minimises risk of airway complications.  PEGs placed intra or preoperatively avoids disturbing the reconstruction postoperatively and can be easily retained and hidden to allow activities of daily living.  PEG complications: 10% had a minor peg complication (wound infection, peritubal discharge/leak) and 8% major complication (accidental removal, tube migration n=1 and abdominal wall abscess). |
| Müller-Richter et al (2017). Nutrition management for head and neck cancer patients improves clinical outcome and survival. | Postoperative early oral diet should aim to preserve swallowing function. States the overall balance should not be sacrificed and the transition to oral diet should ideally be started in the first 24hrs but is often impossible in HNC reconstructive surgery, and in these cases, enteral nutrition must be supported with simultaneous parenteral nutrition. | The transition to oral diet in the first 24hrs is often impossible and enteral nutrition must be supported with simultaneous parenteral nutrition.  Perioperative immunonutrition can have a positive effect on wound healing, reduce infections, fistula formation, complications, LOS and costs in HNC even when compliance is poor. | States a rapid switch to oral intake if swallowing is possible, supplemented with parenteral nutrition.  States goals include sufficient postoperative supplementation with the aim to achieve an energy intake of 30-35kcal/kg/ body weight/day (references ESPEN guidelines for cancer and surgery) and protein intake of 1.2-2g/kg body weight/day to promote would healing and muscle loss (references ESPEN for PN and ASPEN guidelines for critically ill). | Individualised nutrition plans should be determined by the dietitian with regular monitoring of nutritional status.  Adherence / adjustment to nutrition plans should be conducted weekly during the perioperative phase. | Perioperative immunonutrition can reduce infections, fistula formation, complications, hospital stay and costs.  Refeeding syndrome can occur in malnourished patents due to under nutrition supplementation. |
| Yamaguchi et al (2024). Effect of oral intake initiation-establishment interval on hospital stay after oral cancer surgery. | Mean duration from surgery to initiation of oral intake was 15 days (typical of delayed feeding). For the short period group this was 15.1 ± 5.1 days and for the long period group this was 16.8 ± 6.2 days (no significant difference). | Infers all patients had NGT tube feeding postoperatively (but not clear when this was inserted), and that some patients had gastrostomy if they were not able to meet their nutritional requirements orally/ achieve established oral intake. | Dysphagia rehabilitation involved eating after direct training and gradually increasing the meals from 1 to 3. Direct training comprised the use of food, posture, and texture modified diet. Indirect training (for patients who could not commence oral intake) was strength-based and could lead to direct training/meals. Amount of oral intake depended on functional ability.  Period from surgery to established oral intake was significantly quicker for short (16.2 ± 5.3 days) than long group (26.1 ± 8.7 days). Authors report early oral feeding does not necessarily lead to early established oral intake - average period between initiation of oral intake and established oral intake was 3 days (1.1 ± 0.5 days for the early group and 9.3 ± 5.5 days for the late group), significantly longer in older patients (4.5 days, which may be due to reduced muscle mass disuse atrophy and deterioration of oral function / strength).    Criteria for NGT removal: 100% required nutrition calculated (Harris-Benedict 1918 formula) could be consumed orally. If unable to establish oral intake, gastrostomy was considered. | Dentist specialising in dysphagia rehabilitation evaluated swallowing function using videoendoscopic / VFS examinations and developed the rehabilitation plan based on the assessment. States it is desirable that a specialist with sufficient knowledge and skills oversees dysphagia rehabilitation.  States MDT approach used for achieving established oral intake, especially in older patients. | Shortening LOS not only requires earlier initiation but also early established oral intake.  A significant difference was detected in LOS between short and long period groups, and the period between initiation of oral intake and established oral intake was an independent factor associated with LOS. |
| Wu et al (2022) Timing of oral feeding in patients who have undergone free flap reconstruction for oral cancer. | Title states timing of oral feeding, but this was not an outcome measure (NGT removal time was the outcome measure).  For non-tracheostomy patients, on POD1, patients who were able to swallow saliva were allowed a sip of 5ml of water. If no cough was induced, a nurse instructed the patient to have 5ml of water orally every time thirst was experienced. | NGT placed (not clear when these were placed).  NGT removal significantly earlier in the two intervention groups than the 2 control groups. Non tracheostomy control (7.8±3.9days). Non tracheostomy intervention (5±2.3 days). Tracheostomy control (16.2±13 days). Tracheostomy intervention (9.8±5.3days). | Non-tracheostomy control groups received the conventional NGT removal plan (according to the surgeon’s assessment of the wound healing and swallowing ability). For the tracheostomy control patients, after the tracheal tube was removed, NGT removal depended on the wound and swallowing conditions. For the intervention groups, a NGT removal plan was devised to ensure safety and effectiveness with each patients swallowing function being assessed. For non-tracheostomy patients, on POD1, patients able to swallow saliva were allowed a 5ml sip of water. If no cough, patient was instructed to have 5ml of water orally when thirsty, increased to 15ml on POD2 and 30ml on POD3. If no cough or discomfort, the NGT was removed after checking 4 conditions: 1) Watian swallow test level I or II 2) no 'wet voice' after drinking water 3) blood oxygen saturation decreased by <3% within 5 min after oral water and 4) daily oral intake >2000ml. If these conditions were not met, the fluid plan continued until all 4 were met.  States the nurse recorded daily food intake from 1 day before NGT removal and 3 days after NGT removal. Mean daily food intake in the four groups fluctuated between 2204ml on the day of NGT removal and 2272ml 3 days after NGT removal (not statistically significant). All patients had reduced food intake on the day of NGT removal, however, the amount exceeded 2000ml and increased. By POD3, the volume ingested was the same as that before NGT removal.  Estimated requirements were based on national data estimates, where the average weight of a 50-70year old is 59.2-70.6kg. Energy requirements were then calculated using 25-30kcal/kg/day and protein 1-2g/kg/day (Chinese nutritional support for cancer patients’ guidelines). Therefore, a minimum 1480-1765 kcal and 59.2-70.6g protein was estimated for all patients as average, and the 2000ml liquid diet contained 1700kcal and 70g protein, which theoretically should be sufficient. | Safety and effectiveness with each patients swallowing function assessed by nurses specialising in dysphagia or surgeon.  Daily food intake recorded from 1 day before NGT removal to 3 days after NGT removal by nurses.  Clinical decision making for NGT removal is based on treating doctor’s experience; therefore many patients have prolonged NGT. | Early oral intake after surgery does not increase the incidence of wound complications and pneumonia or adversely affect the oral intake of patients, and can help minimise pharyngeal pain, shorten LOS of patients with a tracheostomy and promote salivary secretion, which reduces the risk of wound infection.  Pharyngeal pain (before NGT removal and 24hrs post removal) was evaluated by a nurse using a visual analogue scale. In the tracheostomy groups, pharyngeal pain was significantly lower in the intervention than control groups. States swallowing action will aggravate the pain of the wound and may result in patients limiting their food intake. An NGT removal protocol for such patients can aid safety (by avoiding aspiration). |
| 1 reference, 2 records 1) Kerawala et al (2021). The impact of early oral feeding following head and neck free flap reconstruction on complications and length of stay 2) Singh et al (2021). An urgent need for early oral feeding following head and neck reconstruction with oral defects. | Late feeding group followed a traditional approach (5-day NBM period followed by the introduction of oral intake for which fluids preceded a soft diet by a few days). Early feeding group was followed a philosophical change in unit approach where the aim was to start fluids ± soft diet on POD1. in the early feeding group, 46% commenced fluids and 30% concurrently started solid or semi solid diet. On POD2, 74% fluids, 58% solids. On POD3 94% fluids, 84% solids. Letter to editor advises that the reason for non-uniformity about early feeding was not given i.e. 46% day 1 and no explanation why 54% was not started day 1 and not clear how were patients chosen for early or late feeding as non-randomised study. | States in methods 'where necessary, feeding was supplemented by enteral tube feeding, most commonly in the form of a nasogastric tube'. Not mentioned when this was placed or if any other feeding tubes used | States in methods 'where necessary, feeding was supplemented by enteral tube feeding, most commonly in the form of a nasogastric tube'.  Late feeding group: 5 day NBM then oral intake (fluids preceded a soft diet by a few days). Early feeding group: fluids ± soft, solids or semi-solids diet on the first postoperative day.  Tendency for reduced weight loss in early feeding groups but not significant. | Oral feeding in both groups only commenced if deemed safe by SLT.  Reducing LOS has benefits for patients including physical and psychological. | Early oral feeding was significantly associated with reduced LOS (11.6 versus 20.6 days). In the early feeding group, 26.5% had been discharged by the end of the first week compared to 10% late feeding groups. By the second week, 69% of early feeding group had left hospital compared to 38% late feeding groups. By end of week three, 84.5% of early feeders and 72% late feeders had been discharged.  Previous RT did not increase rate of fistula in early feeding group. Donor site complications reported in 14% of patients and 8% at recipient site which resulted in fistula for 16/18. No significant difference in fistula rates for those with prior RT. No significant differences between the two groups in terms of flap dehiscence or neck fistula rates. |
| Rana et al (2023). Resumption of oral feeding following reconstruction of oral cavity defects using the submental artery perforator flap (SAIPF) | Infers in introduction that early oral feeding is <5 days. Day of oral diet resumption: 11% POD1 9% POD2, 7% POD 3/4 /5/6, 2% POD7, 48% POD>7. Type of oral diet was not specified. Tumour stage (increasing) and defect location (floor of mouth and tongue) were independent predictors for increased time to resume oral feeding following SAIPF. | States feeding tubes used/some patients feeding tube dependent at clinic visit, but not clear what type of tube or when these were placed | 43% discharged on an oral diet and 64% returned to a soft or regular consistency diet by the most recent follow-up appointment and 37% feeding tube dependent. | Introduction states that resuming oral intake following reconstructive surgery is based on multiple factors including surgeon preference (alongside defect size, subsite location, patient comorbidities and others). | Average LOS was 7 days. Increased LOS was correlated with the odds of needing a feeding time at the time of hospital discharge.  In patients undergoing SAIPF some may need tube feeding at the time of discharge, but this is usually temporary. |
| McAfee et al (2019). Enhancing the recovery of head and neck oncology patients through early feeding. | Introduction states a NBM period of 1-2 weeks post resection reduce risks of aspiration and developing a fistula. Authors propose early feeding may improve swallow rehabilitation and reduce LOS; with the units aim to reduce NBM to 2 days or less: 20% tolerated clear fluids by POD1 and 87% by POD2. | - | - | - | Early oral feeding improves utilisation of hospital resources and rehabilitation of patients (swallowing specifically).  All flaps were successful apart from 1, which was not attributed to feeding. |
| Main et al (2017). Early oral intake and length of stay following free flap reconstruction of the oral cavity. | Median postoperative time to oral fluids was 3[1-16] days and to soft diet 6[1-17] days. Median LOS was 9[5-38] days. | - | - | - | Early oral feeding can result in shorter LOS without increased morbidity or additional complications (however, no comparisons were made to traditionally fed groups).  No patients experienced complications relating to early oral feeding. |
| Le et al (2022). Does early oral intake after microvascular free flap reconstruction of the oral cavity lead to increased postoperative complications? | Early oral feeding defined as <5 days.  Late feeding ≥ 5 days.  Not receiving a tracheostomy and having a unilateral neck dissection and no gastrostomy were associated with early oral intake. The early oral intake group had a larger number of reconstructions involving the buccal mucosa and mandible compared to the late group. Both skin paddle area and defect volume were significantly smaller in the early oral intake group than the late group. | 49.6% had a gastrostomy placed during their primary hospitalisation, some were prophylactically placed (avoids delay in hospital discharge associated with procedure scheduling in interventional radiology).  Gastrostomy tubes are placed in anticipation of severe dysphagia following SLT swallow evaluation.  Discussion infers NGT was also used. | - | Initiating oral intake was decided by the surgical team, based on the integrity of the flap/mucosal suture lines and potential for an effective seal separating the oral cavity from the deep cervical lymphadenectomy wound. A bedside swallowing evaluation was then conducted by an SLT prior to initiating oral intake. If there was suspicion of aspiration, a modified barium swallow study was performed. Gastrostomy tubes placed following SLT evaluation. | Early oral intake associated with reduced LOS and fewer lower postoperative complications compared to late oral intake but not when adjusting for covariates. Patients in late oral intake had a higher number of neck infections compared to treatment group during primary hospitalisation. Postoperative complications following discharge (such as exposed hardware/bone, neck infection and OCF) were more frequent in the late feeding group than early group. |
| 1 reference; 2 records: 1) Zhang et al (2022). Effects of personalised swallowing rehabilitation in patients with oral cancer after free flap transplantation: A cluster randomised controlled trial 2) Zhang et al (2024). Effects of personalised oral exercised on swallowing function among patients with oral cancer after free flap transplantation: a cluster randomised controlled trial. | Main outcomes were swallowing function on POD6, 15- and 1-month post operation. Time to first oral intake was not measured but it is inferred the earliest was POD6, where patients receiving rehab before and after meals (consistency not stated) as the first assessment of swallowing took place on this date. 98% dysphagic on POD6. Changes in tongue strength was the only factor by POD30 that was not significantly different between the 2 groups (other factors measured included mouth opening, salivation, lip seal, tongue movement and oral preparation)  Intervention group had personalised swallow rehab 2x per day (before and after meals) which comprised oral sensory stimulation and oral exercise training and post discharge, videos and knowledge of rehab to continue to guide patients through a smartphone App (+control group activities). Control group had routine education related to swallowing (oral hygiene, guiding eating and carrying out effective coughing and conducting supraglottic swallow training). | Nasogastric tube - not mentioned when this was placed  The removal time of the nasogastric tube in the intervention group was 8.38[7–10] days after the operation, which was significantly  shorter than that in the control group, which was 17.76[10–22] days. | Nutritional intake/adequacy not measured in terms of caloric intake.  % weight loss was measured at baseline (POD6, POD15 and 1 month after operation. % weight loss was significantly different between the groups and followed a negative increasing trend and control group showed a positive trend. At POD15 (intervention group -0.20[-1.10-1.09] control, group 2.45[0.91-5.43] and 1 month after operation (intervention group -1.54[-3.40-1.12] and control group 3.33[-0.54 - 4.88] This demonstrated that personalised swallowing rehab after surgery can reduce weight loss and improve nutritional status in postoperative patients.  NGT removal time was significantly shorter in the intervention group (8.38[7-10] days) than control group (17.76[10-20] days) indicating that personalised swallowing rehabilitation can promote earlier NGT removal (no comment about whether oral intake was adequate as a threshold for removal). | States control group had routine nursing and health education related to swallowing.  Personalised swallow rehab was delivered by a swallowing specialist nurse (who is a senior professional in the field of swallowing disorder trained by the Swallowing Disorder Rehabilitation Committee of the Chinese Association of Rehabilitation Medicine). | Personalised swallow rehabilitation can improve swallowing ability, promote early NGT removal and recovery of oral feeding, improve nutritional status and QOL.  Removal criteria of the trial included severe complications, such as flap necrosis or infection after the operation, re-operation or death during the study. Dysphagia was 98.5% at POD6, indicating it is common in patients with oral cancer undergoing free flap. |
| Wu et al (2023). Early swallowing training after free flap surgery in oral cancer: A randomised controlled trial. | Time to oral feeding not measured, just NGT removal time but indicates in discussion that the intervention group had earlier food intake and NGT removal.  Incidence of dysphagia at POD6 was 97.6%, on POD15, 79.7% in control group and 64.5% in intervention group. At 1 month, this was 54.4% control group and 17.1% intervention group. This indicates combined use of oral motor and oral sensory training can support recovery of swallowing function in the early postoperative period. | NGT tubes - not mentioned when these was placed.  NGT indwelling time was significantly lower in the intervention (7.6 ± 3.2 days) than control group (15.9 ± 12 days) and in discussion states that improved swallowing function in the intervention group enabled earlier food intake and earlier NGT removal. | Nutritional intake/adequacy not measured in terms of caloric intake.  % weight loss was measured at baseline (6 days after operation), POD15 and 1 month after surgery. The weight loss rate in the intervention group was significantly lower than control group and showed an upward trend at both time points (POD15 and 1 month), while the control group showed a downward trend (highest on POD15). POD15 (intervention group -0.15[-1.4-1.83] control, group 1.99 [0.58-4.04] and 1 month after operation (intervention group -1.45[-3.25-1.52] and control group 1.73[-0.47-3.95]. | Time to NGT removal recorded by a nurse.  Control group had routine nursing and swallowing related health education.  Swallowing specialist nurse carried out swallowing training/rehab, and assessment of swallowing function. | Early postoperative swallowing training improves postoperative swallow function, nutritional status, QOL and shortens NGT indwelling time.  No significant differences were found in rates of wound complications (tissue flap necrosis, wound infection, wound dehiscence and fistula and pneumonia) between the 2 groups. Patients who developed wound complications or pneumonia within 6 days of surgery were excluded. |
| Nesemeier et al (2017). Evidence-Based Support for Nutrition Therapy in Head and Neck Cancer. | States surgical resection alters anatomy and innervation of swallowing muscles.  For patients with HNC who can safely take oral diet postoperatively, prompt initiation of a calorie and protein rich diet is advocated. | States NGT often placed at the time of surgery and most efficacious in the perioperative when temporary NBM is required for healing or swallowing recovery and is anticipated to occur quickly.  Gastrostomy (pre or Intraop, and even PEG in ICU postop) placed in appropriately selected patients -those with poor preoperative swallowing function and/or anticipated swallowing will be compromised by surgery. Parenteral nutrition is rarely employed (if feeding intolerance or unsafe to feed into the gut). Early EN (within 24hrs), ACG guidelines recommend within 24-48hrs for nutritionally high-risk patients, advancing to goal feeding within 48-72hrs if tolerated or prolonged over 5-7 days if required.  Underfeeding in ICU must be used with cautions in HNSCC. Positive outcomes of immunonutrition vary between studies. No study has demonstrated negative impact. | Barriers to continued NGT include unwillingness of healthcare providers to provide NGT care on an outpatient basis.  An individualised nutritional care approach is mandatory.  Determining the caloric requirements can be via indirect calorimetry (most accurate, rarely available) or standard weight-based equations (may misrepresent actual caloric requirements) including Harris Benedict Equation for energy requirement calculation, with a 1.5 activity factor and 1.5 stress factor (which has no difference to 30kcal/kg/day adjusted body weight). In the HNC population, some even recommend 40-45kcal/kg/day for nutritional repletion after surgery. Protein supplementation was previously recommended 1.2-1.5g/kg/day but recent evidence suggests this should be higher at 2g/kg/day or in the range of 1.5-2.5g/kg/day due to malnutrition and sarcopenia risk. | Review states that decisions regarding alimentation should be made in an MDT setting where SLTs and dietitians can recommend interventions to pre-empt further depletion. Postoperative nutrition strategies should be conceived under a registered dietitian who is familiar with this population and routinely determines caloric, protein and free water goals., feeding schedules and assist in the initiation of parenteral nutrition in select patients + management of refeeding syndrome. Nutritional assessment (PGSGA) should be performed by a trained dietitian in the context of MDT care. | Rehabilitating nutrition is critical for the success of surgical treatment. There are positive outcomes of immunonutrition (reduced LOS, complications, wound infection, leak and fistula). States early EN (within the first 24hrs) has many nutritional and non-nutritional benefits but not specified.  Gastrostomy placement: risks include site infection, leakage around site, local pain, gastric erosion, bowel perforation, intraperitoneal leakage and rarely abdominal wall metastasis via seeding. Despite theoretical risk of damage to an anastomotic site after flap reconstruction, some centres even routinely perform PEG postop. Patients who had a preop gastrostomy had reduced LOS, less weight loss and fewer wound complications than those who had a postop gastrostomy.  NGT risks: laryngeal irritation, persistent gastroesophageal reflux and patient discomfort, increased risk of NGT tube displacement or blockage when compared to PEG.  Nutritionally depleted patients who undergo ablative procedures, (especially after prior RT) are higher risk for developing fistulas and surgical site infections. |
| Khan et al (2021). Early Feeding After Free Flap Reconstruction of Oral Cavity Defects: A Single Arm Non-inferiority Trial (NCT04787939) | Experimental arm: early feeding; liquids by mouth (not specified what type) on the first day after surgery.  Outcome measures include the rate of patients cleared for a diet by the speech language pathology team. | Enteral tube feeding indicated but type/timing not specified. | Outcomes include length of time requiring enteral tube feeds and swallowing evaluation methods measured as stated in background.  Measures/outcomes pertaining to nutritional adequacy not mentioned in trial registry. | SLT to determine the proportion of patients that are cleared for a diet.  Study background infers surgeons as decision makers – ‘Traditionally, surgeons have opted to delay the time to oral feeding because of concern that an early oral diet may stress intraoral suture lines and lead to salivary leaks’  Patient experiences not studied but patient outcome measures are. | Primary outcomes: incidence of OCF or salivary leak (within 30 days of surgery). Secondary outcome measures: incidence of surgical site infection (defined clinically as fever, erythema, swelling or purulent drainage from the surgical site requiring topical or systemic treatment) wound dehiscence, aspiration pneumonia within 30 days.  Secondary outcomes: performance status scale, swallowing-related QOL, tongue and jaw range of motion, rate of oral intake tolerance and LOS. |

^Abbreviations: ESPEN= European Society of Parenteral and Enteral Nutrition; ASPEN = American Society of Parenteral and Enteral Nutrition; ACG = America College of Gastroenterology; FEES= Fibreoptic Endoscopic Evaluation of Swallowing; VFS = videofluroscopy ; BMR = Basal Metabolic Rate; IBW = Ideal Body Weight; BMI = Basal Metabolic Index; IDDSI = International Dysphagia Diet Standardisation Initiative; ND = neck dissection; RA = rectus abdominis flap; LD = latissimus dorsi flap; ALT = anterior lateral thigh flap; FOM = floor of mouth; SLT = Speech and Language Therapist; EN = enteral nutrition; PN = parenteral nutrition; NGT = nasogastric tube; PEG = percutaneous endoscopic gastrostomy; MDT = multidisciplinary team; ORL = otolaryngology; OMFS = oral and maxillofacial surgery; LOS = length of stay; QOL = quality of life; OCF = orocutaneous fistula; PGSGA = patient generated subjective global assessment; POD = postoperative day; ICU = intensive care unit.^
